# P‐Glycoprotein and Breast Cancer Resistance Protein Transporter Inhibition by Cyclosporine and Quinidine on the Pharmacokinetics of Oral Rimegepant in Healthy Subjects

**DOI:** 10.1002/cpdd.1088

**Published:** 2022-03-19

**Authors:** Rajinder Bhardwaj, Julie L. Collins, Joseph Stringfellow, Jennifer Madonia, Matt S. Anderson, Jeri‐anne Finley, David A. Stock, Vladimir Coric, Robert Croop, Richard Bertz

**Affiliations:** ^1^ Certara USA Princeton New Jersey USA; ^2^ Biohaven Pharmaceuticals New Haven Connecticut USA; ^3^ Navitas Data Sciences Pottstown Pennsylvania USA

**Keywords:** cyclosporine, calcitonin gene–related peptide receptor antagonist, interaction, quinidine, rimegepant

## Abstract

Rimegepant (Nurtec ODT)—an orally administered, small‐molecule calcitonin gene–related peptide receptor antagonist indicated for the acute and preventive treatment of migraine—is a substrate for both the P‐glycoprotein and breast cancer resistance protein transporters in vitro. We evaluated the effects of concomitant administration of strong inhibitors of these transporters on the pharmacokinetics of rimegepant in healthy subjects. This single‐center, open‐label, randomized study was conducted in 2 parts, both of which were 2‐period, 2‐sequence, crossover studies. Part 1 (n = 15) evaluated the effect of a single oral dose of 200‐mg cyclosporine, a strong inhibitor of the P‐glycoprotein and breast cancer resistance protein transporters, on the pharmacokinetics of rimegepant 75 mg. Part 2 (n = 12) evaluated the effect of a single oral dose of 600‐mg quinidine, a strong selective P‐glycoprotein transporter, on the pharmacokinetics of rimegepant 75 mg. Coadministration with cyclosporine showed an increase in rimegepant area under the plasma concentration–time curve from time 0 to infinity and maximum observed concentration based on geometric mean ratios (90% confidence intervals [CIs]) of 1.6 (1.49‐1.72) and 1.41 (1.27‐1.57), respectively, versus rimegepant alone. Coadministration with quinidine showed an increase in rimegepant area under the plasma concentration–time curve from time 0 to infinity and maximum observed concentration geometric mean ratios (90% CIs) of 1.55 (1.40‐1.72) and 1.67 (1.46‐1.91), respectively, versus rimegepant alone. Strong P‐glycoprotein inhibitors (cyclosporine, quinidine) increased rimegepant exposures (>50%, <2‐fold). In parts 1 and 2, rimegepant coadministration was well tolerated and safe. The similar effect of cyclosporine and quinidine coadministration on rimegepant exposure suggests that inhibition of breast cancer resistance protein inhibition may have less influence on rimegepant exposure.

Migraine is a chronic neurologic disease characterized by recurrent attacks of headache associated with nausea, vomiting, photophobia, and phonophobia.^1^ Pharmacotherapy for migraine can be acute or preventive, and the armamentarium includes analgesics, nonsteroidal anti‐inflammatory drugs, 5‐hydroxytryptamine receptor agonists, ergot alkaloids, beta‐adrenergic blocking agents, calcium channel blockers, anticonvulsants, and calcitonin gene–related peptide receptor small‐molecule antagonists and monoclonal antibodies.^2^ In practice, because patients with migraine often use multiple medications,^3^ clinically significant drug interactions can limit their utility. For orally administered medications, interaction potential is frequently determined by the effects of the cytochrome P450 (CYP) isoenzyme system and efflux transporters (eg, P‐glycoprotein [P‐gp], breast cancer resistance protein [BCRP]) on drug metabolism.^4^, ^5^

Rimegepant orally disintegrating tablet (ODT) (Nurtec ODT, Biohaven Pharmaceuticals, Inc., New Haven, Connecticut), formerly known as BMS‐927711, is an orally administered, small‐molecule calcitonin gene–related peptide receptor antagonist.^6^, ^7^ Based on demonstrated efficacy and safety in multiple clinical trials,^8^, ^9^, ^10^, ^11^, ^12^, ^13^, ^14^, ^15^ rimegepant is the first migraine medication to receive indications for the acute and preventive treatment of migraine.^16^ Following sublingual administration of rimegepant ODT under fasting conditions, median time to maximum plasma concentration (t_max_) is 1.5 hours, and the absolute oral bioavailability is ≈64%.^14^, ^17^ In vitro studies have shown that rimegepant is primarily metabolized by CYP3A4; reaction phenotyping studies with selective chemical inhibitors and human liver microsomes, as well as with recombinant human CYP enzymes, demonstrated that metabolism of rimegepant was primarily mediated via CYP3A4.^6^, ^7^ CYP2C9 was also implicated in the metabolism of rimegepant in vitro; however, because the influence of CYP2C9 allelic variation did not significantly influence rimegepant metabolism, CYP2C9 was considered not to be a significant mediator of metabolism in vivo.^7^ Rimegepant is a substrate of the P‐gp and BCRP transporters in vitro, but it is not a substrate of the organic anion transporting polypeptides 1B1 and 1B3.^7^

In phase 1 clinical drug‐drug interaction studies (unpublished), coadministration of rimegepant 75 mg with a 200‐mg dose of itraconazole (a strong CYP3A4 and P‐gp inhibitor^18^) once daily for 7 consecutive days to normal, healthy adults increased area under the plasma concentration–time curve (AUC) by 400% and maximum observed concentration (C_max_) by 40%, whereas coadministration of rimegepant 75 mg with a 400 mg dose of fluconazole (a moderate CYP3A inhibitor with no known P‐gp inhibition potential^19^, ^20^, ^21^) once daily for 8 consecutive days increased AUC by 80% with no change in C_max_. Since CYP3A4 inhibitors usually inhibit P‐gp, characterizing the extent to which rimegepant interactions are attributable to CYP3A4 inhibition from those due to transporter inhibition will further facilitate the safe and appropriate use of rimegepant in individuals with migraine. This study was designed to assess the effect of cyclosporine (a nonselective inhibitor of P‐gp and BCRP^22^, ^23^) on the pharmacokinetics (PK) of rimegepant 75 mg in healthy subjects and, if necessary, the effect of quinidine (a specific, strong P‐gp inhibitor^24^) on rimegepant PK.

## Methods

### Ethics

This clinical trial was conducted in accordance with the ethical principles of Good Clinical Practice, the International Conference on Harmonization Harmonized Tripartite Guideline, and all local laws and regulations. Before any study‐related procedures were undertaken, investigators obtained independent ethics committee approval of the protocol and study‐related materials by Advarra Institutional Review Board (Aurora, Ontario, Canada), and subjects provided written informed consent.

### Conduct

This phase 1, open‐label, randomized, single‐dose, 2‐way crossover, 2‐part, drug‐drug interaction study was conducted at a single center (Syneos Health, Québec, Canada). Subjects were screened within 28 days preceding administration of study medication. In part 1, fasted subjects were randomly assigned to a single dose of rimegepant 75 mg ODT or a single dose of rimegepant 75 mg ODT coadministered with cyclosporine 200 mg (2 × 100‐mg capsules). After a washout period of at least 14 days, subjects initially randomized to rimegepant alone received rimegepant coadministered with cyclosporine, and subjects initially randomized to rimegepant‐cyclosporine crossed over to receive rimegepant alone. Following the completion of part 1, an interim PK analysis and review of data were performed. Part 2 was to be conducted only if the rimegepant AUC from time 0 to infinity (AUC_0‐inf_) ratio of geometric means in part 1 was increased by >50% when coadministered with cyclosporine. In part 2, fasted subjects were randomly assigned to a single dose of rimegepant 75 mg ODT or a single dose of rimegepant 75 mg ODT coadministered with quinidine 600 mg (2 × 300‐mg tablets). After a washout period of at least 7 days, subjects initially assigned to rimegepant alone received rimegepant coadministered with quinidine, and those who were initially assigned to rimegepant‐quinidine received rimegepant alone.

### Subjects

Eligible subjects were healthy nonsmokers (no use of tobacco or nicotine products within 3 months before screening) aged ≥18 and ≤55 years with a body mass index between 18.5 and 30 kg/m^2^ and weight of at least 50 kg for men and at least 45 kg for women. They had to have no clinically significant history of neurologic, endocrine, cardiovascular, pulmonary, hematologic (eg, neutropenia), immunologic, psychiatric, gastrointestinal, renal, hepatic, or metabolic disease and a score of 0 on the Sheehan Suicidality Tracking Scale^25^ (S‐STS) at screening. Women were required to have a negative serum or urine pregnancy test with a minimum sensitivity 25 IU/L or equivalent units of human chorionic gonadotropin at screening and day −1. Women of childbearing potential who were sexually active with a nonsterile male partner were required to use acceptable contraceptive methods throughout the study and for 60 days after the last dose of the study drug. Men who were not vasectomized for at least 6 months before the first dose of study drug and who were sexually active with a nonsterile female partner were required to use acceptable contraceptive methods throughout the study and for 90 days after the last dose of the study treatment. Men, including those who had been vasectomized, with a pregnant partner were required to use a condom from the first dose and for 90 days after the last dose of the study treatment.

Subjects with any clinically significant deviation from normal in physical examination, vital signs, electrocardiogram, or clinically significant laboratory determinations were also excluded, as were those who had any medical condition or had used medications that were likely to affect the PK profile of the study drug or subject safety at any time during the study.

The complete inclusion and exclusion criteria are available in the study protocol (Figure S1).

### Treatments

In part 1, subjects received 1 of the following treatments under fasting conditions: 1 oral dose of rimegepant 75 mg ODT (Catalent Pharma Solutions, Swindon, UK) or 1 oral dose of rimegepant 75 mg ODT coadministered with cyclosporine 200 mg (Novartis Pharmaceuticals Canada Inc., Dorval, Quebec, Canada). In part 2, subjects received 1 of the following treatments under fasting conditions: 1 oral dose of rimegepant 75 mg ODT alone or 1 oral dose of rimegepant 75 mg ODT coadministered with quinidine 600 mg (Sandoz Inc., West Princeton, New Jersey). In parts 1 and 2, the interval between treatments could not exceed 2 minutes.

For each treatment, subjects were confined from the morning of day −1 until after the 24‐hour postdose blood draw on day 2. Subjects returned for all subsequent blood draws. No food was allowed from at least 10 hours before dosing until at least 4 hours after dosing. Meals were standardized and similar in composition between periods. Except for water given with cyclosporine and quinidine, no fluids were allowed from 1 hour before dosing until 1 hour after dosing. Water was provided ad libitum at all other times. Subjects were required to remain seated and avoid lying down or sleeping for the first 4 hours after drug administration, but failure to comply with these requirements did not constitute a deviation from the protocol if it was medically necessary, required for procedures, or to go to the bathroom. When appropriate, subjects were accompanied by a staff member while walking.

### Assessments

#### Pharmacokinetics

In each period, 20 blood samples for PK assessment were collected at the following time points: before dosing; 5, 10, 20, 30, 40, and 50 minutes after dosing; and 1, 1.5, 2, 2.5, 3, 4, 5, 6, 8, 12, 24 (day 2), 48 (day 3), and 72 (day 4) hours after dosing. The total volume of blood drawn for the whole study, including that collected for eligibility and safety purposes, did not exceed 195 mL per subject. Plasma samples were assayed for rimegepant using ultra‐performance liquid chromatography with tandem mass spectrometry detection (LC‐MS/MS). The quantifiable range was 10 to 5000 ng/mL. Plasma samples containing human ethylenediaminetetraacetic acid (EDTA) K2 plasma as an anticoagulant were processed using automated protein precipitation before LC‐MS/MS analysis. The internal standard was rimegepant‐^13^C_2_d_4_. The validation was performed using an API 5000 LC/MS/MS system with Analyst software, version 1.6.3 (SCIEX, Framingham, Massachusetts). The pump flow was isocratic, with a flow rate set at 0.600 mL/min. The column was an Acquity UPLC BEH C18, 50×2.1 mm, 1.7 μm (Waters Corporation, Framingham, Massachusetts), which was maintained at a temperature of 60°C and contained Milli‐Q type water/acetonitrile with ammonium acetate and acetic acid. The mass‐to‐charge ratios monitored for rimegepant were 535.4 for the precursor ion and 256.1 for the product ion, whereas for the internal standard, they were 541.4 for the precursor ion and 256 for the product ion. The precision of rimegepant calibration standards ranged from 3.28% to 6.96%; the between‐run accuracy bias ranged from −2.49 to 3.39%. The accuracy and precision of quality control samples was ≤15% (≤20% at the lower limit of quantitation), and calibration curves for the LC‐MS/MS bioanalytical assay were within acceptable limits. Incurred sample reanalysis was carried out on ≈10% of randomly selected samples. More than two thirds of the incurred reanalyses of samples were within acceptable limits (20% deviation).

#### Safety

Safety was assessed using adverse events (AEs) that occurred from signing informed consent through 4 days after the last study drug administration, physical examination, body measurements, and the S‐STS. Safety was also assessed through vital signs (blood pressure, heart rate, respiratory rate, oral temperature), and electrocardiography. In part 2, cardiac telemetry was recorded continuously from ≈10 hours before dosing until 6 hours after dosing in each period (8 hours after dosing in subjects receiving quinidine with an increased QT interval at the 6‐hour assessment).

Safety laboratory assessments included a QuantiFERON‐TB test (Hilden, Germany); pregnancy tests (urine and serum); drug, alcohol, and cotinine screen; hematology; biochemistry; coagulation (in case of abnormal liver function tests); serology; and urinalysis.

### Sample Size

In total, 32 healthy adults were planned. Based on data from previous studies (unpublished), the intrasubject coefficient of variation for rimegepant was expected to be ≈27% for both AUC and C_max_. With a 27% coefficient of variation and the assumption that the true ratio was the target ratio to determine initiation of part 2 of 1.5, there was at least 90% power to detect a statistically significant difference in AUC, ranging from 92.5% with 12 subjects to 98.1% with 16 subjects.

### Randomization and Blinding

Subjects were randomly assigned to 1 of the 2 sequences, and each treatment was administered according to the 2‐period, 2‐sequence, block randomization scheme produced for each study part separately. The randomization code was not available to study personnel until the clinical and analytical phases of each study part had been completed.

Because this was an open‐label study, blinding and unblinding were not used.

### Statistical Methods

The following PK parameters were calculated by standard noncompartmental methods for rimegepant using Phoenix WinNonlin version 8.0 (Certara, Princeton, New Jersey): AUC from time 0 to the last observed concentration (AUC_0‐t_), AUC_0‐inf_, C_max_, residual area, t_max_, elimination half‐life (t_1/2el_), terminal elimination rate constant, apparent body clearance, and apparent volume of distribution.

The effects of cyclosporine (part 1) or quinidine (part 2) on the single‐dose PK of rimegepant were evaluated using general linear model procedures in SAS (SAS Institute, Cary, North Carolina). In these evaluations, analysis of variance (ANOVA) model was performed on untransformed t_max_, terminal elimination rate constant, and t_1/2el_ and on log‐transformed AUC_0‐t_, AUC_0‐inf_, and C_max_ at the alpha level of .05. The model included treatment, sequence, and period as fixed effects, and subject nested within sequence as a random effect. Intra‐ and intersubject coefficient of variation were estimated. The ratios of geometric and arithmetic means in part 1 (rimegepant‐cyclosporine:rimegepant) and part 2 (rimegepant‐quinidine:rimegepant) and 90% CIs for the ratios of geometric and arithmetic means, based on least squares means from the ANOVA of the log‐transformed data, were calculated for AUC_0‐t_, AUC_0‐inf_, and C_max_. The 90% CIs for the ratios of AUC_0‐inf_ and C_max_ were used to quantify the extent of drug interaction. Because subjects in part 1 were dosed in 2 groups, the statistical model was modified to reflect the multigroup nature of the study, with group, sequence, sequence*group, period (group) treatment, and treatment*group interaction as fixed effects and subject (sequence*group) as a random factor. Summaries for concentrations and PK parameters and ANOVA drug interaction analyses were performed for subjects who completed both treatment periods (SAS PROC GLM in parts 1 and 2; a sensitivity analysis was performed in parallel for subjects who completed at least 1 treatment period (SAS PROC MIXED) in parts 1 and 2.

Concentration values below the limit of quantitation were set to 0. Samples with no reportable value before dosing were replaced by “0.00” and set to missing for tabulations, graphical representations, and calculations. Estimates for missing data were not extrapolated or interpolated. For the PK analysis, only observed data were used, except when concentration values were below the limit of quantitation.

Safety data were summarized but not subjected to inferential analysis.

## Results

### Subjects

In part 1, 16 fasted subjects were dosed: 15 subjects received a single dose of rimegepant 75 mg ODT, and 16 subjects received a single dose of rimegepant 75 mg ODT coadministered with cyclosporine 200 mg. One subject in the rimegepant group was not dosed and was discontinued from the study by the investigator due to an elevation in creatine kinase >2× the upper limit of normal. Fifteen (94%) subjects completed part 1 of the study (Figure S2).

In part 2, 15 fasted subjects received a single dose of rimegepant 75 mg ODT, and 14 fasted subjects received a single dose of rimegepant 75 mg ODT coadministered with quinidine 600 mg. In total, 2 of 15 subjects discontinued due to AEs, 1 in the rimegepant group due to an AE of sinus tachycardia and 1 in the rimegepant‐quinidine group due to a false‐positive test result for severe acute respiratory syndrome coronavirus 2. Altogether, 1 of 15 subjects discontinued for vomiting within 4 hours after coadministration of rimegepant with quinidine; the subject was not included in the PK summary analysis but was included in the safety population. Among subjects who were dosed in part 2, 12 of 14 completed the study (Figure S2).

The demographics of the populations in both parts of the study are presented in Table 1.

**Table 1:** Demographics and Baseline Characteristics

	Part 1	Part 2
	N = 16	N = 15
Age, y, mean (SD)	43 (10)	43 (9)
Sex, n (%)		
Female	7 (44)	5 (33)
Male	9 (56)	10 (66)
White race, n (%)	16 (100)	15 (100)
Weight, kg, mean (SD)	72 (12)	78 (13)
Body mass index, kg/m^2^, mean (SD)	25 (3)	26 (3)

Abbreviation: SD, standard deviation.

### Pharmacokinetics

**Figure 1:**
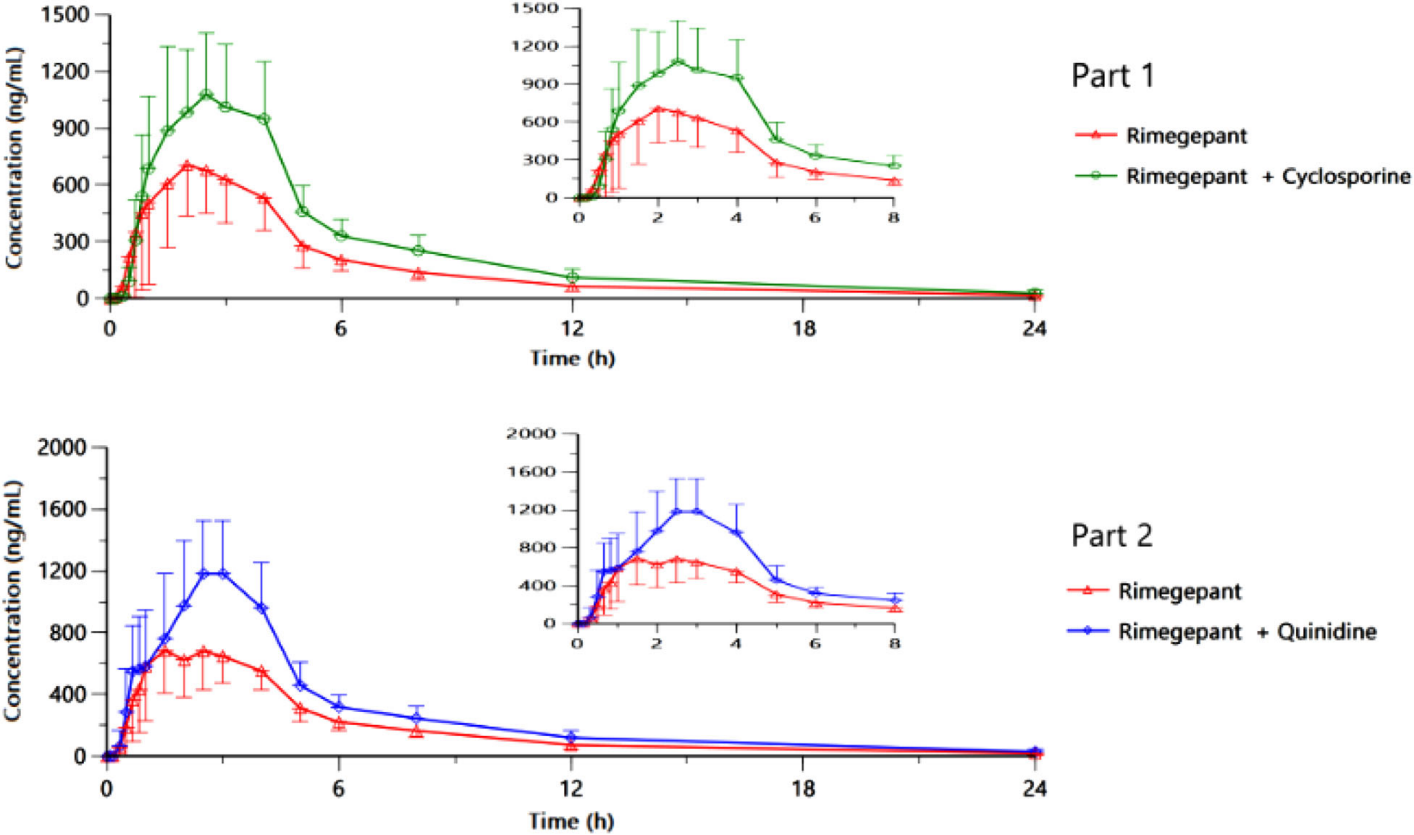
Mean (SD) plasma rimegepant concentration‐time profiles through 24 hours^a^ for rimegepant 75 mg ODT alone or with cyclosporine 200 mg (part 1) and rimegepant alone or with quinidine 600 mg (part 2). ODT, orally disintegrating tablet; SD, standard deviation. ^a^Results were truncated at 24 hours; values below the limit of quantitation were set to 0 for the calculation of means.

Higher mean rimegepant plasma concentrations were observed during coadministration of rimegepant with cyclosporine than with rimegepant alone (Figure 1, Part 1). Among subjects who completed both periods in part 1, cyclosporine increased AUC_0‐inf_ and C_max_ of a single dose of rimegepant 75 mg ODT by 60.1% and 41%, respectively (Table 2). The geometric mean t_1/2el_ was similar between treatments (within 5%), and median t_max_ was 2.5 hours when coadministered with cyclosporine versus 2 hours when administered alone (Table 3).

**Table 2:** Geometric Least Squares Mean Ratios and 90% CIs of Rimegepant 75 mg ODT AUC and C_max_ When Administered Alone, With Cyclosporine 200 mg (Part 1), or With Quinidine 600 mg (Part 2)

Part 1				
Rimegepant 75 mg ODT Alone or With Cyclosporine 200 mg (N = 15)				
	Geometric Least Squares Mean		Rimegepant + Cyclosporine vs Rimegepant	
	Rimegepant + Cyclosporine	Rimegepant	Ratio^a^ (%)	90% CI (%)
AUC_0‐t_, ng • h/mL	6561.18	4092.08	160.34	149.15‐172.36
AUC_0‐inf_, ng • h/mL	6574.36	4106.19	160.11	149.04‐172.00
C_max_, ng/mL	1162.70	824.41	141.03	126.98‐156.64

Part 2				
Rimegepant 75 mg ODT Alone or With Quinidine 600 mg (N = 12)				
	Geometric Least Squares Mean		Rimegepant + Quinidine vs Rimegepant	
	Rimegepant + Quinidine	Rimegepant	Ratio^a^ (%)	90% CI (%)
AUC_0‐t_, ng • h/mL	6814.74	4396.19	155.01	139.59‐172.15
AUC_0‐inf_, ng • h/mL	6826.00	4408.49	154.84	139.42‐171.96
C_max_, ng/mL	1329.08	796.57	166.85	145.82‐190.92

Abbreviations: AUC, area under the plasma concentration–time curve; AUC_0‐inf_, AUC from time 0 to infinity; AUC_0‐t_, AUC from time 0 to the last observed concentration; C_max_, maximum observed concentration; ODT, orally disintegrating tablet.

^a^ Geometric mean.

**Table 3:** Rimegepant Pharmacokinetic Parameters Following a Single Oral Dose of Rimegepant ODT 75 mg Alone and Coadministered With Cyclosporine 200 mg or Quinidine 600 mg^a^

	N	C_max,_ ng/mL	AUC_0‐t_, ng • h/mL	AUC_0‐inf_, ng • h/mL	t_max_, h	t_½el_, h	CL/F, L/h
Part 1							
Rimegepant	15	862.04 (31.63)	4253.05 (31.92)	4267.03 (31.83)	1.99 (0.66‐3.00)	7.59 (19.20)	19.26 (31.84)
Rimegepant + cyclosporine	15	1240.82 (31.76)	6772.48 (24.64)	6785.53 (24.60)	2.49 (1.00‐3.99)	7.32 (16.31)	11.77 (28.24)
Part 2							
Rimegepant	12	828.98 (23.97)	4524.94 (22.75)	4537.38 (22.70)	1.50 (0.67‐3.99)	8.56 (20.20)	17.39 (24.68)
Rimegepant + quinidine	12	1356.76 (25.29)	6940.30 (22.51)	6952.08 (22.49)	2.77 (0.67‐3.99)	7.24 (23.18)	11.27 (21.58)

Abbreviations: AUC, area under the concentration‐time curve; AUC_0‐t_, AUC from time 0 to the last observed concentration; AUC_0‐inf_, AUC from time 0 to infinity; C_max_, maximum observed concentration; CL/F, apparent clearance; ODT, orally disintegrating tablet; t_½el_, elimination half‐life; t_max_, time to maximum concentration.

^a^ Values are arithmetic mean (percent coefficient of variation), except t_max_, which is median (range).

Higher mean rimegepant plasma concentrations were observed during coadministration of rimegepant with quinidine compared with rimegepant alone (Figure 1, Part 2). Quinidine increased C_max_ and AUC_0‐inf_ of a single dose of rimegepant 75 mg ODT by 66.9% and 54.8%, respectively (Table 2). The geometric mean t_1/2el_ between treatments was within 20%, and median t_max_ was 2.8 hours when coadministered with quinidine and 1.5 hours when administered alone.

### Safety

Overall, 3 of 16 subjects in part 1 and 14 of 15 subjects in part 2 experienced at least 1 treatment‐emergent AE (TEAE). The most frequently reported TEAEs (≥2 subjects in each part) were QT prolongation (12 subjects in the rimegepant‐quinidine group); nausea (4 subjects in the rimegepant‐quinidine group; 2 subjects in the rimegepant‐cyclosporine group, and 1 subject in the rimegepant group); dizziness and asthenia (3 subjects each in the rimegepant‐quinidine group); and soft feces, gastrointestinal pain, and hot flush (2 subjects each in the rimegepant‐quinidine group). All other TEAEs were reported in 1 subject each. All TEAEs were mild or moderate in severity. All TEAEs resolved by the end of study, except for the TEAE of sinus tachycardia in part 2.

No serious AEs were reported. No clinically meaningful changes from baseline in vital signs or S‐STS were identified. Twelve of 15 subjects had clinically significant QT prolongation during coadministration of rimegepant with quinidine; all the events were mild and resolved on the same day without treatment.

## Discussion

This study evaluated the effects of strong inhibitors of the P‐gp transporter on the PK of rimegepant in healthy subjects. Coadministration with cyclosporine increased rimegepant AUC_0‐inf_ and C_max_ geometric least squares mean ratios by 60% and 41%, respectively, compared with rimegepant alone. The >50% increase in AUC triggered part 2 of the study, in which rimegepant was coadministered with the selective P‐gp inhibitor quinidine, which increased rimegepant AUC_0‐inf_ and C_max_ geometric least squares mean ratios by 55% and 67%, respectively, versus rimegepant alone. These data show that strong P‐gp inhibitors, such as cyclosporine, quinidine, amiodarone, carvedilol, dronedarone, lapatinib, propafenone, ranolazine, and verapamil,^26^ have the potential to increase the exposure of rimegepant by more than 50% but less than 2‐fold. Previously, single and 2‐week multiple daily oral doses of rimegepant up to 600 mg have been shown to be well tolerated.^27^, ^28^ The recommended dose of rimegepant for the acute treatment of migraine is 75 mg repeated up to once every 24 hours. The increased exposure of rimegepant due to coadministration of cyclosporine or quinidine is similar to a rimegepant dose of 112.5 mg but less than a rimegepant dose of 150 mg. It is recommended that rimegepant dosing frequency be limited to no more than once every 48 hours when coadministered with a strong P‐gp inhibitor so the average exposure of rimegepant will not exceed that of 75 mg of rimegepant administered once daily.

With respect to CYP3A4 inhibition, the effects of cyclosporine on rimegepant AUC can be contextualized by comparing them with the results of a previously published study of cyclosporine. Coadministration of cyclosporine with oral midazolam (a more sensitive CYP3A4 substrate than rimegepant with no P‐gp involvement^18^) increased midazolam AUC by 46% with no meaningful change in C_max_,^29^ while coadministration of fluconazole increased rimegepant AUC by 80% with no change in C_max_. In light of these findings, the results of the present study suggest that the increased rate and extent of rimegepant absorption can be attributed to inhibition of intestinal efflux transporters (P‐gp and/or BCRP) rather than to inhibition of CYP3A4 by cyclosporine.

The results of the rimegepant‐quinidine interaction study (part 2), where a higher rate and extent of rimegepant absorption were demonstrated by increased rimegepant C_max_, suggest that inhibition of P‐gp by quinidine occurs in the intestine. The similarity of the results following coadministration of rimegepant with quinidine or cyclosporine indicate that the effects of BCRP inhibition on rimegepant exposure were inconsequential. The lack of evidence for quinidine inhibition of CYP3A4 distinguishes it from cyclosporine and further demonstrates that the observed effects on rimegepant exposure by cyclosporine and quinidine were primarily due to inhibition of P‐gp.

A single oral dose of rimegepant 75 mg ODT administered alone or coadministered with cyclosporine or quinidine was well tolerated. Although the most frequently reported TEAE was QT prolongation, this event occurred exclusively in quinidine‐treated subjects and was anticipated, as quinidine is known to prolong the QT interval in a dose‐dependent fashion.^30^

## Conclusions

Strong P‐gp inhibitors increased rimegepant exposure by >50% but <2‐fold; BCRP inhibition influence on rimegepant exposure was minimal.

## Conflicts of Interest

RB, JLC, JM, JF, DAS, VC, and RC are employed by and own stock/stock options in Biohaven Pharmaceuticals. MSA, JS, and RB were paid consultants to Biohaven Pharmaceuticals.

## Funding

This study was supported by Biohaven Pharmaceuticals.

## Supporting information

Figure 1

Figure 2
