# Supplementary material for: P‐Glycoprotein and Breast Cancer Resistance Protein Transporter Inhibition by Cyclosporine and Quinidine on the Pharmacokinetics of Oral Rimegepant in Healthy Subjects
Source: Clin Pharmacol Drug Dev. 2022 Mar 19;11(7):889–97. doi: 10.1002/cpdd.1088 (PMC9311059; doi:10.1002/cpdd.1088)
Supplement: Supplementary file 1 — Figure 1 [file CPDD-11-889-s002.pdf]

PROTOCOL 200133

Biohaven Pharmaceuticals, Inc. study number: BHV3000-122

IND Number: 109886

A PHASE 1, OPEN-LABEL, RANDOMIZED, TWO-WAY CROSSOVER, TWO-PART STUDY TO EVALUATE THE EFFECTS OF CYCLOSPORINE (PART I) AND QUINIDINE (PART II) ON THE PHARMACOKINETICS OF RIMEGEPANT 75 mg ORALLY DISINTEGRATING TABLET IN HEALTHY SUBJECTS

Contract Research Organization:
Syneos Health Clinique inc.
(« Syneos Health »)

2500, rue Einstein
Québec (Québec)
Canada, G1P 0A2
Tel.: 1-418-527-4000

Sponsor:
Biohaven Pharmaceuticals, Inc.

215 Church Street
New Haven, CT 06510
USA
Tel.: 203-404-0410

CONFIDENTIAL

This document is strictly confidential. It was developed for Biohaven Pharmaceuticals, Inc. by Syneos Health and must not be disclosed to a third party, with the exception of regulatory agencies and study audit personnel without the consent of either Syneos Health or the Sponsor. This document is copyrighted in favor of Syneos Health and cannot be reproduced, modified, or adapted, in part or in total, without prior written approval by Syneos Health.

Protocol Historical File

Version number	Brief description/summary of changes	Date
Final Draft	Version submitted to the Food and Drug Administration (FDA)	20-MAY-2020
Final	Version submitted to the Independent Ethics Committee (IEC)	28-JUL-2020

Signature Page

Sponsor

Biohaven Pharmaceuticals, Inc.
215 Church Street
New Haven, CT 06510
USA
Tel.: 203-404-0410

Signature Page

Contract Research Organization (CRO)

Investigator:

I have carefully read this study protocol and agree that it contains all necessary information required to conduct this study. I agree to conduct the study according to this protocol (including any amendments) and in accordance with the clinical site's Standard Operating Procedures (SOPs), ICH Good Clinical Practice (GCP), all other applicable regulations, and the recommendations laid down in the most recent version of the Declaration of Helsinki.

1 FACILITIES AND RESPONSIBLE STAFF

1.1 Clinical Research Facilities

This study will be conducted by Syneos Health at the following facility:

2500, rue Einstein
Québec (Québec), Canada, G1P 0A2
Tel.: 1-418-527-4000

Screening and/or visits may also be performed at the Montréal Syneos Health facility:

5160, boul. Décarie, suite 800
Montréal (Québec), Canada, H3X 2H9
Tel.: 1-514-485-7500

1.2 Biomedical Laboratory Facilities

Biomedical laboratory testing will be performed by the following laboratories:

Biron Medical Laboratory inc.
4105-F, boul. Matte
Brossard (Québec), Canada, J4Y 2P4
Tel.: 1-514-866-6146

Syneos Health
2500, rue Einstein
Québec (Québec), Canada, G1P 0A2
Tel.: 1-418-527-4000

Syneos Health
5160, boul. Décarie, suite 800
Montréal (Québec), Canada, H3X 2H9
Tel.: 1-514-485-7500

QuantiFERON®-TB test will be performed by the following laboratory:

Les Laboratoires BNK inc.
500 boul. Cartier O., suite 205
Laval (Québec), Canada, H7V 5B7
Tel.: 1-450-688-4432

If another biomedical laboratory is used, this will be documented and annexed to the protocol.

1.3 Clinical Pharmacology and Regulatory Affairs

Syneos Health
2500, rue Einstein
Québec (Québec), Canada, G1P 0A2
Tel.: 1-418-527-4000

1.4 Bioanalytical Facility

Syneos Health
2500, rue Einstein
Québec (Québec), Canada, G1P 0A2
Tel.: 1-418-527-4000
Clark V. Williard, B.S.
Executive Director, Mass Spectrometry Operations

TABLE OF CONTENTS

1 FACILITIES AND RESPONSIBLE STAFF 5
1.1 CLINICAL RESEARCH FACILITIES 5
1.2 BIOMEDICAL LABORATORY FACILITIES 5
1.3 CLINICAL PHARMACOLOGY AND REGULATORY AFFAIRS 6
1.4 BIOANALYTICAL FACILITY 6
2 SYNOPSIS OF PROTOCOL 9
3 LIST OF ABBREVIATIONS AND DEFINITION OF TERMS 16
4 SCHEDULE OF EVENTS\ 19
5 INTRODUCTION 21
5.1 BACKGROUND INFORMATION ON RIMEGEPANT 21
5.2 BACKGROUND INFORMATION ON CYCLOSPORINE 21
5.3 BACKGROUND INFORMATION ON QUINIDINE 22
5.4 RATIONALE FOR STUDY DESIGN 23
5.5 RATIONALE FOR INITIATION OF PART II 24
5.6 RATIONALE FOR DOSE SELECTION 24
5.7 RATIONALE FOR STUDY POPULATION 25
6 OBJECTIVES 26
6.1 PRIMARY OBJECTIVES 26
6.2 SECONDARY OBJECTIVES 26
7 STUDY DESIGN 26
8 STUDY POPULATION 27
8.1 SAMPLE SIZE 27
8.2 INCLUSION CRITERIA 27
8.3 EXCLUSION CRITERIA 28
9 CLINICAL PROCEDURES 31
9.1 SCREENING PROCEDURES 32
9.2 CONFINEMENTS, VISITS AND WASHOUT 32
9.3 RANDOMIZATION AND BLINDING 32
9.4 STUDY TREATMENTS 33
9.5 DRUG SUPPLIES AND ACCOUNTABILITY 33
9.6 DRUG ADMINISTRATION 34
9.7 STUDY RESTRICTIONS 34
9.8 SAMPLE COLLECTION AND PROCESSING 36
9.9 SAFETY MONITORING 36
9.10 STUDY EXIT/EARLY TERMINATION PROCEDURES 39
9.11 DATA COLLECTION AND EVALUATION 40
9.12 SUBJECT WITHDRAWAL AND REPLACEMENT 40
9.13 ADVERSE EVENTS 41
9.14 PREGNANCY 45
9.15 REPORTABLE DISEASE 45
9.16 PREMATURE TERMINATION OF THE STUDY 46
10 ANALYTICAL METHODOLOGY 46
11 PHARMACOKINETIC AND STATISTICAL ANALYSES 46
11.1 PHARMACOKINETIC PARAMETERS 47
11.2 ANALYSIS POPULATIONS 47

11.3 STATISTICAL ANALYSES....48
12 FINAL REPORT....49
13 REGULATORY CONSIDERATIONS AND QUALITY ASSURANCE....49
13.1 INDEPENDENT ETHICS COMMITTEE APPROVAL OF PROTOCOL AND OTHER STUDY DOCUMENTS....49
13.2 COMPLIANCE....49
13.3 QUALITY ASSURANCE PROGRAM....50
13.4 AUDITS, INSPECTIONS AND MONITORING....50
14 CONFIDENTIALITY AND RETENTION OF STUDY RECORDS....51
15 REFERENCES....52

2 SYNOPSIS OF PROTOCOL

**Project No.:**	200133<br>Biohaven Pharmaceuticals, Inc. study number: BHV3000-122
**Study Title:**	A PHASE 1, OPEN-LABEL, RANDOMIZED, TWO-WAY CROSSOVER, TWO-PART STUDY TO EVALUATE THE EFFECTS OF CYCLOSPORINE (PART I) AND QUINIDINE (PART II) ON THE PHARMACOKINETICS OF RIMEGEPANT 75 mg ORALLY DISINTEGRATING TABLET IN HEALTHY SUBJECTS
**Study Drug:**	Rimegepant (BHV-3000) Orally Disintegrating Tablets (ODT), 75 mg<br>Trademark: Nurtec ODT
**Study Phase and Type:**	Phase 1 – Drug-Drug Interation (DDI)
**Primary Objectives:**	Part I: To evaluate the effect of single-dose administration of cyclosporine on the single-dose pharmacokinetics (PK) of rimegepant.<br>Part II: To evaluate the effect of single-dose administration of quinidine on the single-dose PK of rimegepant.
**Secondary Objectives:**	Part I: To assess the safety and tolerability of a single-dose of rimegepant co-administered with cyclosporine in healthy subjects.<br>Part II: To assess the safety and tolerability of a single-dose of rimegepant co-administered with quinidine in healthy subjects.
**Study Design:**	This will be a single center, Phase 1, open-label, randomized, two-part study to be conducted as follows:<br>Part I:<br>Two-period, two-sequence, crossover DDI study to evaluate the effect of cyclosporine, administered as 2 x 100 mg capsules, on the PK of rimegepant administered as 1 x 75 mg ODT in healthy subjects under fasting conditions.<br>Interim PK analysis and review of data will be performed following Part I completion. Part II will be conducted if rimegepant $AUC_{0\text{-}inf}$ ratio of geometric means in Part I is increased by more than 50% when co-administered with cyclosporine.<br>Part II:<br>Two-period, two-sequence, crossover DDI study to evaluate the effect of quinidine, administered as 2 x 300 mg tablets, on the PK of rimegepant administered as 1 x 75 mg ODT in healthy subjects under fasting conditions.
**Subjects:**	Up to 16 male or female volunteers in each part of the study, for a total of up to 32 subjects.
**Inclusion Criteria:**	Subjects must meet all of the following criteria to be included in the study:<br>1) Male or female, non-smoker (no use of tobacco or nicotine products within 3 months prior to screening), ≥18 and ≤55 years of age, with body mass index (BMI) >18.5 and <30.0 kg/m^2 and body weight ≥50.0 kg for males and ≥45.0 kg for females.<br>2) Healthy as defined by:<br>a) the absence of clinically significant illness. Subjects who have experienced vomiting within 24 hours pre-dose will be carefully evaluated for upcoming illness/disease. Inclusion pre-dosing is at the discretion of the Investigator.

	b) the absence of clinically significant history of neurological, endocrine, cardiovascular, pulmonary, hematological (e.g., neutropenia), immunologic, psychiatric, gastrointestinal, renal, hepatic, and metabolic disease.<br>3) Subject's score on the Sheehan Suicidality Tracking Scale (S-STS) at screening must be 0.<br>4) Females must have a negative serum or urine pregnancy test (minimum sensitivity 25 IU/L or equivalent units of human chorionic gonadotropin [HCG]) at screening and Day -1.<br>5) Females must not breastfeed.<br>6) Females of childbearing potential who are sexually active with a non-sterile male partner (sterile male partners are defined as men vasectomized for at least 6 months prior to the first dosing) must be willing to use one of the following acceptable contraceptive methods throughout the study and for 60 days after the last dosing:<br>a) simultaneous use of intra-uterine contraceptive device without hormone release system placed at least 4 weeks prior to the first dosing, and condom for the male partner;<br>b) simultaneous use of diaphragm or cervical cap with intravaginally applied spermicide and male condom for the male partner, started at least 21 days prior to the first dosing.<br>7) Male subjects who are not vasectomized for at least 6 months prior to the first dosing and who are sexually active with a non-sterile female partner (sterile female partners include post-menopausal females defined as amenorrheic for at least 12 consecutive months prior to the first dosing and surgically sterile females who have undergone hysterectomy, bilateral oophorectomy, or tubal ligation at least 6 months prior to the first dosing) must be willing to use one of the following acceptable contraceptive methods throughout the study and for 90 days after the last dosing:<br>a) simultaneous use of a male condom and, for the female partner, hormonal contraceptives or intra-uterine contraceptive device used since at least 4 weeks prior to the first dosing;<br>b) simultaneous use of a male condom and, for the female partner, a diaphragm or cervical cap with intravaginally applied spermicide.<br>8) Male subjects (including men who have had a vasectomy) with a pregnant partner must agree to use a condom from the first dosing and for 90 days after the last dosing.<br>9) Male subjects must be willing not to donate sperm for at least 90 days after the last dosing.<br>10) Subjects must be able to understand the nature of the study, agree to comply with the prescribed dosage regimens, and communicate to study personnel about adverse events (AEs) and concomitant medication use, as applicable.<br>11) Subjects must sign and date the IEC-approved informed consent obtained prior to the conduct of any study activities.<br>12) Subjects must be willing to remove dentures or mouth piercing, if applicable, at the time of dosing.
**Exclusion Criteria:**	Subjects to whom any of the following applies will be excluded from the study:<br>***Medical History and Concurrent Diseases***<br>1) Current diagnosis of viral hepatitis or a history of liver disease.

	2) Significant history of seizure disorder other than a single childhood febrile seizure (e.g., epilepsy).<br>3) Current or recent (within 3 months of study drug administration) clinically significant gastrointestinal disease that may interfere with drug absorption.<br>4) Gastrointestinal surgery that interferes with physiological absorption and motility (i.e., gastric bypass, duodenectomy, or gastric banding).<br>5) History of significant alcohol abuse or regular use of alcohol within 6 months prior to screening (more than 14 units for males or 7 units for females of alcohol per week [1 unit = 150 mL of wine, 360 mL of beer, or 45 mL of 40% alcohol]).<br>6) History of significant drug abuse within 6 months prior to screening, or use of soft drugs (such as marijuana) within 3 months or hard drugs (such as cocaine, phencyclidine [PCP], crack, opioid derivatives including heroin, and amphetamine derivatives) within 1 year prior to screening.<br>7) History of allergic reactions to rimegepant, cyclosporine, quinidine, or other related drugs, or to any excipient in the formulations.<br>8) History of anaphylaxis, documented hypersensitivity reaction, or clinically significant reaction to any drug.<br>9) Clinically significant history of depression or suicidal thoughts within 6 months prior to screening.<br>10) Use of medications for the timeframes specified below, with the exception of medications exempted by the Investigator on a case-by-case basis if they are judged unlikely to affect the PK profile of the study drug or subject safety (e.g., topical drug products without significant systemic absorption):<br>a) prescription medications within 14 days prior to the first dosing;<br>b) over-the-counter products (including acetaminophen-containing products) and natural health products (including herbal remedies, homeopathic and traditional medicines, probiotics, food supplements such as vitamins, minerals, amino acids, essential fatty acids, and protein supplements used in sports) within 14 days prior to the first dosing, with the exception of the occasional use of ibuprofen;<br>c) live attenuated vaccine within 1 month prior to the first dosing or planned vaccination during the course of the study;<br>d) injectable or implantable hormonal contraceptive agent within 2 months prior to the first dosing;<br>e) depot injection or implant of any other drug within 3 months prior to the first dosing;<br>f) any drug known to induce or inhibit hepatic drug metabolism, including St. John's wort, within 1 month prior to the first dosing.<br>11) Donation of plasma within 7 days prior to dosing. Donation or loss of blood (excluding volume drawn at screening) of 50 mL to 499 mL of blood within 30 days, or more than 499 mL within 56 days prior to the first dosing.<br>12) Participation in a clinical research study involving the administration of an investigational or marketed drug or device within 30 days prior to the first dosing, administration of a biological product in the context of a clinical research study within 90 days prior to the first dosing, or concomitant participation in an investigational study involving no drug or device administration.<br>13) For subjects participating in Part I of the study:

   a) History of latent or active tuberculosis or exposure to endemic areas within 8 weeks prior to QuantiFERON®-TB testing performed at screening.
   b) Positive QuantiFERON®-TB test indicating possible tuberculosis infection.
   c) History of clinically significant opportunistic infection (e.g., invasive candidiasis or pneumocystis pneumonia).
   d) Serious local infection (e.g., cellulitis, abscess) or systemic infection (e.g., septicemia) within 3 months prior to screening.
   e) Clinically significant history of uncontrolled hypertension.
   f) History of active malignancy.
   g) History of psoriasis, except if mild psoriasis (small number of minor plaques requiring no treatment or only intermittent topical steroids).

14) For subjects participating in Part II of the study:
   a) History of myasthenia gravis, optic neuritis, prolongation of the QT interval, ventricular arrhythmias, thrombocytopenia, atrial fibrillation or flutter, uncorrected hypokalemia, or bradycardia.
   b) Have previously been dosed in Part I of the study.

Physical and Laboratory Test Findings

15) Any abnormal laboratory test results deemed clinically significant by the Investigator or positive test during medical screening.
16) Positive test for human immunodeficiency virus (HIV), hepatitis B surface antigen (HBsAg), or hepatitis C virus (HCV) during medical screening.
17) Evidence of organ dysfunction or any clinically significant deviation from normal on physical examination, vital signs, 12-lead electrocardiogram (ECG), or clinical laboratory determinations beyond what is consistent with the target population.
18) Any of the following laboratory parameters above the upper limit of normal (ULN) values at screening or Day -1: alkaline phosphatase (ALP), aspartate aminotransferase (AST), alanine aminotransferase (ALT), gamma glutamyl-transpeptidase (GGT), direct bilirubin, indirect bilirubin, and total bilirubin. Only abnormal values between 1-1.5x ULN may be repeated once for confirmation to below ULN.
19) Any of the following abnormalities on 12-lead ECG or blood pressure (BP) at screening, confirmed by repeat:
   a) PR (PR interval) $\geq$210 msec.
   b) QRS (QRS complex) $\geq$120 msec.
   c) QTcF (Fridericia's corrected QT interval) $>$450 msec.
   d) Sitting (for at least 5 minutes) systolic BP $>$140 mmHg, confirmed by repeat.
   e) Sitting (for at least 5 minutes) diastolic BP $>$90 mmHg, confirmed by repeat.
20) Any of the following abnormal laboratory test values at screening or Day -1:
   a) Hemoglobin $<$128 g/L for males and $<$115 g/L for females.
   b) Hematocrit $<$0.37 L/L for males and $<$0.32 L/L for females.
   c) Total white blood cell (WBC) $<3.0 \times 10^9$/L.
   d) Platelet count $<100 \times 10^9$/L.

	e) Neutrophils <1.4 x 10^9/L and <1.0 x 10^9/L for Afro-American volunteers.<br>f) Creatine phosphokinase (CPK) >2x ULN.<br>21) Positive urine drug screen, urine cotinine test, or alcohol breath test at screening or Day -1.<br>22) Presence of fever (body temperature >37.6°C) (e.g., a fever associated with a symptomatic viral or bacterial infection) within 2 weeks prior to the first dosing.<br>23) Presence of orthodontic braces or orthodontic retention wires, or any physical finding in the mouth or tongue that would be likely to interfere with successful completion of the dosing procedure.<br>24) Inability to be venipunctured and/or tolerate catheter venous access.<br>25) Inability or difficulty swallowing tablets or capsules.<br>26) Any reason which, in the opinion of the Investigator, would prevent the subject from participating in the study.
**Screening Procedures:**	Demographic data, medical and medication histories, complete physical examination, body measurements, S-STS, vital signs (BP, heart rate [HR], respiratory rate [RR], and oral temperature [OT]), 12-lead ECG, hematology, biochemistry, serology (HIV, HBsAg, and HCV), urinalysis, urine pregnancy test, urine drug screen, urine cotinine test, and alcohol breath test.<br>For subjects in Part I, a QuantiFERON®-TB test will also be performed, unless documented results are available within 2 months preceding dosing. In any case, the standard tuberculosis medical history screening questionnaire will be administered to each subject.
**Confinements, Visits and Washout:**	For each period, subjects will be confined from the morning of Day -1 until after the 24-hour post-dose blood draw on Day 2. Subjects will come back for all subsequent blood draws.<br>For Part I, there will be a washout period of at least 14 days between doses.<br>For Part II, there will be a washout period of at least 7 days between doses.
**Study Treatments:**	<u>Part I</u>:<br>In each period, subjects will receive one of the following treatments:<br>Treatment A: 1 x 75 mg rimegepant ODT to be held under the tongue (sublingual administration) until fully dissolved then swallowed without water, administered under fasting conditions.<br>Treatment B: 1 x 75 mg rimegepant ODT co-administered with 2 x 100 mg cyclosporine capsules under fasting conditions.<br><u>Part II</u>:<br>In each period, subjects will receive one of the following treatments:<br>Treatment A: 1 x 75 mg rimegepant ODT to be held under the tongue (sublingual administration) until fully dissolved then swallowed without water, administered under fasting conditions.<br>Treatment C: 1 x 75 mg rimegepant ODT co-administered with 2 x 300 mg quinidine (as sulfate) tablets under fasting conditions.<br>No food will be allowed from at least 10 hours before dosing until at least 4 hours post-dose.<br>Except for water given for Treatments B and C, no fluids will be allowed from 1 hour before dosing until 1 hour post-dose.

**Study Restrictions:**	Subjects will be asked to refrain from using products that may potentially affect their safety and/or the PK profile of the study drug. Main study restrictions include:<br>• Live attenuated vaccine from 1 month prior to the first dosing and during the study;<br>• Prescription medication from 14 days prior to the first dosing until after the last PK blood sample collection of the study;<br>• Over-the-counter products (including acetaminophen-containing products) from 14 days prior to the first dosing until after the last PK blood sample collection of the study;<br>• Natural health products from 14 days prior to the first dosing until after the last PK blood sample collection of the study;<br>• St. John's wort from 1 month prior to the first dosing until after the last PK blood sample collection of the study;<br>• Food or beverages containing grapefruit, starfruit, pomegranate, pineapple, or pomelo from 14 days prior to the first dosing after the last PK blood sample collection of the study;<br>• Food or beverages containing xanthine derivatives or xanthine-related compounds or energy drinks from 48 hours prior to dosing until after the last PK blood sample collection of each period;<br>• Alcohol-based products from 24 hours prior to admission until after the last PK blood sample collection of each period;<br>• Food containing poppy seeds within 24 hours prior to admission of each period.<br>For safety reasons, subjects will be required to remain seated and avoid lying down or sleeping for the first 4 hours after drug administration.<br>Subjects will be advised not to travel to countries where tuberculosis is endemic during the study and for 14 days after the last dosing.<br>Subjects will be advised to avoid close contact with people with bacterial or viral infections (e.g., flu, gastroenteritis, etc.) during the study.
**Sample Collection for PK Analyses:**	A total of 20 blood samples will be collected in each period for rimegepant measurement: pre-dose and 0.083, 0.167, 0.333, 0.5, 0.667, 0.833, 1, 1.5, 2, 2.5, 3, 4, 5, 6, 8, 12, 24 (Day 2), 48 (Day 3), and 72 (Day 4) hours post-dose.
**Safety Monitoring:**	Physical examination:<br>Brief physical examination: on Day -1 in each period.<br>Vital signs:<br>OT: on Day -1, before dosing on Day 1, and approximately 24, 48, and 72 hours post-dose in each period.<br>BP and HR for Part I: before dosing on Day 1 and approximately 2, 4, 6, 24, 48, and 72 hours post-dose in each period.<br>BP and HR for Part II: before dosing on Day 1 and approximately 1, 2, 4, 6, 8, 12, 24, 48, and 72 hours post-dose in each period.<br>ECG:<br>12-lead ECG for Part I: before dosing on Day 1 in each period.<br>12-lead ECG for Part II: approximately 24 hours post-dose in each period.<br>Cardiac telemetry:

	Continuous cardiac telemetry for Part II: from approximately 10 hours pre-dose until 6 hours post-dose in each period. 12-lead ECG will be extracted before dosing on Day 1 and approximately 1, 2, 4, and 6 hours post-dose in each period.<br>Laboratory assessments:<br>Serum pregnancy test, urine drug screen, urine cotinine test, and alcohol breath test: on Day -1 in each period.<br>Hematology*, biochemistry**, and urinalysis: on Day -1 in each period.<br>*Haptoglobin and reticulocyte count will be performed in case of decrease in hemoglobin levels below the lower limit of normal (LLN).<br>**Coagulation tests will be performed in case of abnormal liver function tests (LFT).<br>Medical surveillance and AE monitoring:<br>Subjects will be monitored throughout the study by the clinical staff for AEs. In each period, a physician will be on site for drug administration and until 4 hours post-dose, and available on call for the remainder of the study.
**Study Exit/Early Termination Procedures:**	Brief physical examination, S-STS, vital signs (BP, HR, RR, and OT), 12-lead ECG, hematology (including haptoglobin and reticulocyte count in case of decrease in hemoglobin levels below the LLN), biochemistry (including coagulation in case of abnormal LFT), urinalysis, urine pregnancy test, concomitant medications, and AE monitoring.
**Analytical Method:**	The Bioanalytical Division of Syneos Health will analyze rimegepant in plasma samples using a validated method.
**PK Parameters:**	AUC_{0-t}, AUC_{0-inf}, C_{max}, Residual area, T_{max}, $T_{½ el}$, and K_{el}.
**Statistical Analyses:**	Details of statistical analyses will be developed in a Statistical Analysis Plan (SAP).<br>PK Analyses:<br>Using general linear model (GLM) procedures in Statistical Analysis System (SAS), analysis of variance (ANOVA) will be performed on untransformed T_{max}, K_{el}, and $T_{½ el}$ and on ln-transformed AUC_{0-t}, AUC_{0-inf}, and C_{max} at the alpha level of 0.05. The ratio of geometric means (B/A for Part I and C/A for Part II) and 90% confidence interval (CI) for the ratio of geometric means, based on least-squares means from the ANOVA of the ln-transformed data, will be calculated for AUC_{0-t}, AUC_{0-inf}, and C_{max}.<br>The 90% CI for the ratios of AUC_{0-inf} and C_{max} will be used to quantify the extent of drug interaction.<br>Interim PK analysis and review of data will be performed following Part I completion. Part II will be conducted if rimegepant AUC_{0-inf} ratio of geometric means in Part I is increased by more than 50% when co-administered with cyclosporine.<br>Safety and Tolerability Analyses:<br>Safety and tolerability will be evaluated through the assessment of AEs (i.e., seriousness, severity, relationship to the study medication, outcome, duration, and management), vital signs, 12-lead ECG, clinical laboratory parameters and physical examination. Treatment-emergent adverse events (TEAEs) will be tabulated by treatment. AEs will be coded using the latest version of the Medical Dictionary for Regulatory Activities (MedDRA). Safety and tolerability data will be reported using descriptive statistics.

3 LIST OF ABBREVIATIONS AND DEFINITION OF TERMS

AE	Adverse Event
ALP	Alkaline Phosphatase
ALT	Alanine Aminotransferase
ANOVA	Analysis of Variance
AST	Aspartate Aminotransferase
AUC	Area Under the Curve
BCRP	Breast Cancer Resistance Protein
BMI	Body Mass Index
BP	Blood Pressure
CFR	Code of Federal Regulations
CGRP	Calcitonin Gene-Related Peptide
CI	Confidence Interval
C_{max}	Maximal Observed Concentration
CPK	Creatine Phosphokinase
CRF	Case Report Form
CRO	Clinical Research Organization
CTA	Clinical Trial Application
CV	Coefficient of Variation
CYP	Cytochrome P450
DDI	Drug-Drug Interaction
ECG	Electrocardiogram
EDC	Electronic Data Capture
FDA	Food and Drug Administration
GCP	Good Clinical Practice
GGT	Gamma Glutamyl-Transpeptidase
GLM	General Linear Model
GLP	Good Laboratory Practice
GMP	Good Manufacturing Practice
HBsAg	Hepatitis B Surface Antigen
HCV	Hepatitis C Virus
HCG	Human Chorionic Gonadotrophin

HEENT	Head, Eyes, Ears, Nose, and Throat
HIV	Human Immunodeficiency Virus
HR	Heart Rate
ICF	Informed Consent Form
IB	Investigator Brochure
ICH	International Council for Harmonisation of Technical Requirements for Pharmaceuticals for Human Use
IEC	Independent Ethics Committee
INR	International Normalized Ratio
ISCV	Intra-Subject Coefficient of Variation
IV	Intravenous
K_{el}	Terminal Elimination Rate Constant
kg	Kilogram
L	Liter
LFT	Liver Function Test
LIMS	Laboratory Information Management System
LLN	Lower Limit of Normal
Max	Maximum
MedDRA	Medical Dictionary for Regulatory Activities
mg	Milligram
Min	Minimum
mL	Milliliter
mmHg	Millimeter Mercury
MRHD	Maximum Recommended Human Dose
msec	Millisecond
NOL	No Objection Letter
ODT	Orally Disintegrating Tablet
OT	Oral Temperature
PCP	Phencyclidine
P-gp	P-glycoprotein
PK	Pharmacokinetic(s)
PR	PR interval
aPTT	activated Partial Thromboplastin Time

PT	Prothrombin Time
QA	Quality Assurance
QC	Quality Control
QTcF	Fridericia's corrected QT interval
RR	Respiratory Rate
SAE	Serious Adverse Event
SAP	Statistical Analysis Plan
SAS	Statistical Analysis System
SD	Standard Deviation
SOP	Standard Operation Procedure
S-STS	Sheehan Suicidality Tracking Scale
$T_{½ el}$	Terminal Elimination Half-Life
TEAE	Treatment-emergent Adverse Event
T_{max}	Time When the Maximal Concentration is Observed
ULN	Upper Limit of Normal
WBC	White Blood Cell

4 SCHEDULE OF EVENTS\

PROCEDURE	Screening	PART I and PART II Periods 1 and 2				Study Exit/Early Termination
		D-1	D1	D2	D3-4	
Informed Consent	X					
Demographic Data	X					
Medical and Medication Histories	X					
Review and Monitoring of AEs and Concomitant Medications		X	X	X	X	X
Physical Examination	X[1]	X[1]				X[1]
Body Measurements (Weight, Height, BMI)	X					
S-STS	X					X
QuantiFERON®-TB Test	X[2]					
Vital Signs (BP, HR, RR, OT)	X	X[3]	X[3]	X[3]	X[3]	X
12-lead ECG	X		X[4]	X[4]		X
Cardiac Telemetry		X[5]	X[5]			
Hematology	X	X[6,7]				X[7]
Biochemistry	X	X[6]				X
Coagulation		X[8]				X[8]
Serology (HIV, HBsAg, HCV)	X					
Urinalysis	X	X[6]				X
Urine Pregnancy Test	X					X
Serum Pregnancy Test		X[9]				
Urine Drug Screen	X	X[9]				
Urine Cotinine Test	X	X[9]				
Alcohol Breath Test	X	X[9]				
Confinement		X[10]	X[10]	X[10]		
Drug Administration			X			
PK Blood Samples			X[11]	X[11]	X[11]	

1 A complete physical examination will be performed at screening. A brief physical examination will be performed on Day -1 in each period and at study exit.

2 For subjects in Part I, a QuantiFERON®-TB test will be performed at screening, unless documented results are available within 2 months preceding dosing. In any case, the standard tuberculosis medical history screening questionnaire will be administered to each subject.

3 OT: on Day -1, before dosing on Day 1, and approximately 24, 48, and 72 hours post-dose in each period.

BP and HR for Part I: before dosing on Day 1 and approximately 2, 4, 6, 24, 48, and 72 hours post-dose in each period.

BP and HR for Part II: before dosing on Day 1 and approximately 1, 2, 4, 6, 8, 12, 24, 48, and 72 hours post-dose in each period.

4 12-lead ECG for Part I: before dosing on Day 1 in each period.

12-lead ECG for Part II: approximately 24 hours post-dose in each period.

5 Continuous cardiac telemetry for Part II: from approximately 10 hours pre-dose until 6 hours post-dose in each period. 12-lead ECG will be extracted before dosing on Day 1 and approximately 1, 2, 4, and 6 hours post-dose in each period.

6 Laboratory assessments (i.e., hematology, biochemistry, and urinalysis) will be performed on Day -1 in each period.

7 Haptoglobin and reticulocyte count will be performed in case of decrease in hemoglobin levels below the LLN.

8 Coagulation tests (PT/INR and aPTT) will be performed only in case of abnormal LFT.

9 Serum pregnancy test, urine drug screen, urine cotinine test, and alcohol breath test will be performed on Day -1 in each period.

10 Subjects will be confined from the morning of Day -1 until after the 24-hour post-dose blood draw on Day 2.

11 PK blood samples: pre-dose and 0.083, 0.167, 0.333, 0.5, 0.667, 0.833, 1, 1.5, 2, 2.5, 3, 4, 5, 6, 8, 12, 24 (Day 2), 48 (Day 3), and 72 (Day 4) hours post-dose.

5 INTRODUCTION

5.1 Background Information on Rimegepant

Rimegepant is a calcitonin gene-related peptide (CGRP) receptor antagonist indicated for the treatment of acute migraine with or without aura in adults. The recommended dose is 75 mg taken orally. The maximum dose in a 24-hour period is 75 mg. Adverse events associated with rimegepant are listed in the Prescribing Information.[1]

CGRP is an endogenous 37 amino acid peptide contained within pain signaling nociceptive afferents and is thought to play a causal role in migraine. Multiple lines of clinical evidence point to a role for CGRP in migraine pathophysiology: 1) serum levels of CGRP are elevated during migraine in man, 2) treatment with anti-migraine drugs returns CGRP levels to normal coincident with pain relief, and 3) intravenous (IV) CGRP infusion produces lasting pain in non-migraineurs and migraineurs. Treatment with a CGRP receptor antagonist is thought to relieve migraine by 1) blocking CGRP-induced neurogenic vasodilation (returning dilated intracranial arteries to normal), 2) halting the cascade of CGRP induced neurogenic inflammation (which leads to peripheral sensitization), and possibly 3) inhibiting the central relay of pain signals from trigeminal nerve to the caudal trigeminal nucleus.[2]

Following sublingual administration of rimegepant ODT under fasting conditions, peak plasma concentrations are reached with a median T_{max} of 1.5 hours. The absolute oral bioavailability of rimegepant in the fasted state is approximately 64%. Rimegepant plasma exposures increase over the single dose range of 25 mg to 900 mg. Rimegepant is approximately 96% bound to human plasma proteins.[2]

Rimegepant is primarily metabolized by cytochrome P450 (CYP) 3A4 and to a lesser extent by CYP2C9. Rimegepant is primarily eliminated in unchanged form (~77% of the dose) with no major metabolites (i.e., >10%) detected in plasma. Following oral administration of [^{14}C]-rimegepant to healthy male subjects, 78% of the total radioactivity was recovered in feces and 24% in urine. Unchanged rimegepant is the major single component in excreted feces (42%) and urine (51%). The elimination half-life of rimegepant is approximately 11 hours in healthy subjects.[1]

5.2 Background Information on Cyclosporine

Cyclosporine is a potent immunosuppressive agent indicated for the prevention of graft rejection following solid organ or bone marrow transplantation, in the treatment of severe psoriasis and severe active rheumatoid arthritis, and for steroid dependent and steroid resistant nephrotic syndrome due to glomerular diseases. The recommended oral dose ranges from 2.5 to 15 mg/kg/day, depending on the condition treated. Adverse events associated with cyclosporine are listed in the Product Monograph.[3]

Cyclosporine strongly suppresses cell mediated immunity and is therefore highly effective in preventing allograft rejection.[3] The effectiveness of cyclosporine results from specific and reversible inhibition of immunocompetent lymphocytes in the G0-and G1-phase of the cell cycle. T-lymphocytes are preferentially inhibited. The T-helper cell is the main target, although the

T-suppressor cell may also be suppressed. Cyclosporine also inhibits lymphokine production and release including interleukin-2.[4]

Following oral administration of cyclosporine capsules under fasting conditions, peak blood concentrations are reached with a median T_{max} of 1.5 hours.5 The relationship between the administered dose and exposure is linear within the therapeutic dose range. Cyclosporine is distributed largely outside the blood volume. In the blood, 33 to 47 % is present in plasma, 4 to 9% in lymphocytes, and 41 to 58% in erythrocytes. In plasma, approximately 90% is bound to proteins, mostly lipoproteins.[3,4]

Cyclosporine is extensively metabolized by the CYP 3A enzyme system in the liver, and to a lesser degree in the gastrointestinal tract, and the kidney. At least 25 metabolites have been identified from human bile, feces, blood, and urine. The biological activity of the metabolites and their contributions to toxicity are considerably less than those of the parent compound.[4] The major route of elimination of cyclosporine is through the bile. Less than 1% of an administered dose of cyclosporine is excreted in the bile as parent drug. More than 44% of a cyclosporine dose appears in the bile as metabolites when measured by RIA. Enterohepatic recirculation of parent drug is thus very low.[3] The elimination half-life of cyclosporine is approximately 19 hours in healthy subjects.[5]

5.3 Background Information on Quinidine

Quinidine is an antimalarial schizonticide and an antiarrhythmic agent with class 1A activity indicated for the treatment of life-threatening *Plasmodium falciparum* malaria and for the conversion of atrial fibrillation/flutter, reduction of frequency of relapse into atrial fibrillation/flutter, and suppression of ventricular arrhythmias. The initial recommended dosage is 200 mg to 400 mg every six hours, depending on the condition treated. Adverse events associated with quinidine are listed in the Drug Label Information.[6]

In patients with malaria, quinidine acts primarily as an intra-erythrocytic schizonticide, with little effect upon sporozites or upon pre-erythrocytic parasites. Quinidine is gametocidal to *Plasmodium vivax* and *P. malariae,* but not to *P. falciparum*. In cardiac muscle and in Purkinje fibers, quinidine depresses the rapid inward depolarizing sodium current, thereby slowing phase-0 depolarization and reducing the amplitude of the action potential without affecting the resting potential. In normal Purkinje fibers, it reduces the slope of phase-4 depolarization, shifting the threshold voltage upward toward zero. The result is slowed conduction and reduced automaticity in all parts of the heart, with increase of the effective refractory period relative to the duration of the action potential in the atria, ventricles, and Purkinje tissues. Quinidine also raises the fibrillation thresholds of the atria and ventricles, and it raises the ventricular defibrillation threshold as well. Quinidine's actions fall into class 1A in the Vaughan-Williams classification.[6]

The absolute bioavailability of quinidine from quinidine sulfate tablets is about 70%, but this varies widely (45 to 100%) between patients. The less-than-complete bioavailability is the result of first-pass metabolism in the liver. Peak serum levels generally appear about 2 hours after dosing. At concentrations of 2 to 5 mg/L (6.5 to 16.2 µmol/L), the fraction of quinidine bound to

plasma proteins (mainly to α1-acid glycoprotein and to albumin) is 80 to 88% in adults and older children.[6]

Most quinidine is eliminated hepatically via the action of CYP3A4; there are several different hydroxylated metabolites, and some of these have antiarrhythmic activity. The most important of quinidine's metabolites is 3-hydroxyquinidine, serum levels of which can exceed those of quinidine in patients receiving conventional doses of quinidine sulfate. When the urine pH is less than 7, about 20% of administered quinidine appears unchanged in the urine, but this fraction drops to as little as 5% when the urine is more alkaline. Renal clearance involves both glomerular filtration and active tubular secretion, moderated by (pH-dependent) tubular reabsorption. The elimination half-life of quinidine is 6 to 8 hours in adults.[6]

5.4 Rationale for Study Design

Rimegepant is primarily metabolized by the CYP3A4 enzyme and is also a substrate of P-glycoprotein (P-gp) and breast cancer resistance protein (BCRP) efflux transporters. Rimegepant exposures are increased in the presence of a strong or moderate inhibitor of CYP3A4. CYP3A4 inhibitors often show measurable inhibition of P-gp and for this reason, it has been hypothesized that concomitant administration of rimegepant with inhibitors of the P-gp, and/or BCRP transporters may result in a significant increase in rimegepant exposure.[1] However, no specific drug interaction study has yet been conducted to assess the effects of concomitant administration of inhibitors of P-gp and BCRP transporters on the PK of rimegepant.

In accordance with the recommendations provided in the FDA Guidance on *Clinical Drug Interaction Studies – Cytochrome P450 Enzyme- and Transporter-Mediated Drug Interactions, Guidance for Industry (2020)*, to understand the transporter-mediated DDI of the largest possible magnitude for an investigational drug that is a substrate for multiple transporters, an inhibitor of several transporters can be used as the inhibitor in the DDI study. Negative results from this kind of study can rule out the need to further evaluate the drug as a substrate for any of the individual transporters. If the study result is positive, additional studies with more selective inhibitors of specific transporter pathways can help determine the relative contribution of each transporter to the disposition of the substrate drug.[7]

Based on the above-stated guidance and FDA's website on *Drug Development and Drug Interactions: Table of Substrates, Inhibitors and Inducers*, cyclosporine has been selected as a well-established inhibitor of both the P-gp and BCRP transporters. In contrast, quinidine is a more specific clinical inhibitor of the P-gp transporter than cyclosporine and has been selected as an appropriate probe, if necessary, to evaluate the individual contributions of the P-gp and BCRP transporters (by differential) to an overall cyclosporine effect.[7,8]

Therefore, this study is designed to first assess the effect of cyclosporine as an inhibitor of both P-gp and BCRP transporters on the PK of rimegepant ODT in Part I. In the event that the exposure ($AUC_{0\text{-}inf}$) of rimegepant is increased by more than 50% (using ratio of geometric means) when co-administered with cyclosporine, the effect of quinidine on the PK of rimegepant via selective inhibition of the P-gp transporter will then be evaluated in Part II (see section 5.5). The effect of BCRP inhibition on rimegepant may then be inferred from the difference between

the results of Part I and Part II. In addition, in order to reduce inter-subject variability, a crossover randomized design has been chosen for this study.

5.5 Rationale for Initiation of Part II

The effects of CYP3A4 inhibition on rimegepant PK have been investigated using the strong CYP3A4 inhibitor, itraconazole, which demonstrated a ~1.5-fold increase in C_{max} and 4-fold increase in AUC. Clinical study with a moderate CYP3A4 and strong CYP2C9 inhibitor, fluconazole, showed no significant effect on C_{max} and a 1.8-fold increase in AUC.[1] In these studies, C_{max} was less sensitive to change than AUC. For this reason, apparent change in geometric mean AUC alone is the parameter that will determine whether to conduct Part II.

If the cyclosporine-based transporter investigation of Part I were to demonstrate that one or more transporter-mediated processes were contributing to these results, then further investigation of individual transporters would be warranted, only if the increase in AUC were meaningful and quantifiable. Under current labeling, an increase in AUC similar to that seen with fluconazole has resulted in labeling language restricting the frequency of rimegepant dose administration to not more than once every 48 hours in the presence of a moderate CYP3A4 inhibitor.[1] To be consistent with this labeling (AUC <1.8-fold elevation) and to ensure that further clinical transporter investigations are clinically meaningful, Part II will be conducted upon demonstration of an increased AUC ratio of geometric means of >50% (>1.50-fold) in the presence of cyclosporine vs. in the absence of this probe. A determination of 50% or lower increase in the AUC of rimegepant will be interpreted as evidence that adjustment for rimegepant dose frequency is unnecessary in the presence of a P-gp or BCRP inhibitor.

5.6 Rationale for Dose Selection

Rimegepant ODT will be administered once in each period at the recommended therapeutic dose of 75 mg.[1]

Based on information found in the literature, most DDI studies with cyclosporine were conducted in a single-dose fashion using doses ranging from 100 to 600 mg. More specifically, studies that explored the interaction of transporter substrates in the absence and presence of cyclosporine at doses of 200 and 600 mg within the same study demonstrated that P-gp and BCRP substrates showed closely similar responses to inhibition by cyclosporine at these doses.[9,10] Given the immunosuppressive properties of cyclosporine and that both P-gp and BCRP transporters appeared sufficiently inhibited with a dose of 200 mg to appropriately characterize changes in the substrates PK, a single-dose of 200 mg cyclosporine has been selected for Part I of this study.

Literature search showed that both single and multiple doses of quinidine have been used in DDI studies. However, studies that used a single dose of 600 mg quinidine demonstrated that the P-gp transporter was sufficiently inhibited at this dosage to adequately assess the clinical magnitude of drug interactions with P-gp subtrates.[11,12] Given the potential for quinidine to cause cardiac effects, a single-dose of 600 mg quinidine sulfate has been selected for Part II of this study, as it is deemed sufficient to allow a safe assessment of the interaction of rimegepant, if necessary, with the P-gp transporter.

5.7 Rationale for Study Population

A healthy volunteer population has been selected for the study, because healthy subjects with no concomitant diseases who are free of concomitant medications represent a homogenous population, allowing for proper evaluation of the safety, tolerability, and PK of a drug without confounding factors. The chosen doses of rimegepant, cyclosporine, and quinidine have been shown to be well tolerated in this population.

There are no adequate data on the developmental risk associated with the use of rimegepant in pregnant women. In animal studies, oral administration of rimegepant (0, 10, 60, or 300 mg/kg/day) to pregnant rats during the period of organogenesis resulted in decreased fetal body weight and an increased incidence of fetal variations at the highest dose tested (300 mg/kg/day), which was associated with maternal toxicity. Plasma exposures (AUC) at the no-effect dose (60 mg/kg/day) for adverse effects on embryofetal development were approximately 45 times that in humans at the maximum recommended human dose (MRHD) of 75 mg/day. Oral administration of rimegepant (0, 10, 25, or 50 mg/kg/day) to pregnant rabbits during the period of organogenesis resulted in no adverse effects on embryofetal development. The highest dose tested (50 mg/kg/day) was associated with plasma exposures (AUC) approximately 10 times that in humans at the MRHD.[1]

There are no adequate data in pregnant women with the use of cyclosporine. In animal studies, cyclosporine was not teratogenic, but was shown to be both embryo- and feto-toxic in rats and rabbits at 2 to 5 times the human dose. In two published research studies, rabbits exposed to cyclosporine in utero (10 mg/kg/day subcutaneously) demonstrated reduced numbers of nephrons, renal hypertrophy, systemic hypertension, and progressive renal insufficiency up to 35 weeks of age. Pregnant rats which received 12 mg/kg/day of cyclosporine intravenously (twice the recommended human intravenous dose) had fetuses with an increased incidence of ventricular septal defect. These findings have not been demonstrated in other species and their relevance for humans is unknown.[3]

Animal reproductive studies have not been conducted with quinidine and there are no adequate and well-controlled studies in pregnant women. Human placental transport of quinidine has not been systematically studied. In one neonate whose mother had received quinidine throughout her pregnancy, the serum level of quinidine was equal to that of the mother, with no apparent ill effect. The level of quinidine in amniotic fluid was about three times higher than that found in serum. In another case, the levels of quinidine and 3-hydroxyquinidine in cord blood were about 30% of simultaneous maternal levels.[6]

Given the above-mentioned information on the study drugs, only males and non-pregnant, non-lactating females will be included in the study. In addition, females of childbearing potential will be included if they use appropriate methods of contraception.

6 OBJECTIVES

6.1 Primary Objectives

Part I: To evaluate the effect of single-dose administration of cyclosporine on the single-dose PK of rimegepant.

Part II: To evaluate the effect of single-dose administration of quinidine on the single-dose PK of rimegepant.

6.2 Secondary Objectives

Part I: To assess the safety and tolerability of a single-dose of rimegepant co-administered with cyclosporine in healthy subjects.

Part II: To assess the safety and tolerability of a single-dose of rimegepant co-administered with quinidine in healthy subjects.

7 STUDY DESIGN

This will be a single center, Phase 1, open-label, randomized, two-part study to be conducted as follows:

Part I:

Two-period, two-sequence, crossover DDI study to evaluate the effect of cyclosporine, administered as 2 x 100 mg capsules, on the PK of rimegepant administered as 1 x 75 mg ODT in healthy subjects under fasting conditions.

Interim PK analysis and review of data will be performed following Part I completion. Part II will be conducted if rimegepant $AUC_{0\text{-}inf}$ ratio of geometric means in Part I is increased by more than 50% when co-administered with cyclosporine.

Part II:

Two-period, two-sequence, crossover DDI study to evaluate the effect of quinidine, administered as 2 x 300 mg tablets, on the PK of rimegepant administered as 1 x 75 mg ODT in healthy subjects under fasting conditions.

Each part of the study is intended to dose in one group; if, for any reason, any part of the study is dosed in more than one group, all groups will be dosed at the same clinical site and the same protocol requirements and procedures will be followed within each group.

8 STUDY POPULATION

8.1 Sample Size

It is planned to dose up to 16 male or female volunteers in each part of the study, for a total of up to 32 subjects.

Based on data from previous studies, the intra-subject coefficient of variation (ISCV) for rimegepant should be approximately 27% for both AUC and C_{max}.5 Thus, with this expected ISCV and assuming the true ratio is the target ratio to determine initiation of Part II of 1.5, there is at least 90% power to detect a statistically significant difference in AUC, ranging from 92.5% at N=12 to 98.1% at N=16. This sample size is judged sufficient to provide a reliable estimate of the magnitude and variability of the drug interaction in each part of the study.

8.2 Inclusion Criteria

Subjects enrolled in this study will be members of the community at large. The recruitment advertisements may use various media types (e.g., radio, newspaper, the clinical site Web site and volunteer database). Subjects must meet all of the following criteria to be included in the study:

1. Male or female, non-smoker (no use of tobacco or nicotine products within 3 months prior to screening), ≥18 and ≤55 years of age, with BMI >18.5 and <30.0 kg/m^2 and body weight ≥50.0 kg for males and ≥45.0 kg for females.

2. Healthy as defined by:

   a. the absence of clinically significant illness. Subjects who have experienced vomiting within 24 hours pre-dose will be carefully evaluated for upcoming illness/disease. Inclusion pre-dosing is at the discretion of the Investigator.

   b. the absence of clinically significant history of neurological, endocrine, cardiovascular, pulmonary, hematological (e.g., neutropenia), immunologic, psychiatric, gastrointestinal, renal, hepatic, and metabolic disease.

3. Subject's score on the S-STS at screening must be 0.

4. Females must have a negative serum or urine pregnancy test (minimum sensitivity 25 IU/L or equivalent units of HCG) at screening and Day -1.

5. Females must not breastfeed.

6. Females of childbearing potential who are sexually active with a non-sterile male partner (sterile male partners are defined as men vasectomized for at least 6 months prior to the first dosing) must be willing to use one of the following acceptable contraceptive methods throughout the study and for 60 days after the last dosing:

   a. simultaneous use of intra-uterine contraceptive device without hormone release system placed at least 4 weeks prior to the first dosing, and condom for the male partner;

   b. simultaneous use of diaphragm or cervical cap with intravaginally applied spermicide and male condom for the male partner, started at least 21 days prior to the first dosing.

7. Male subjects who are not vasectomized for at least 6 months prior to the first dosing, and who are sexually active with a non-sterile female partner (sterile female partners include post-menopausal females defined as amenorrheic for at least 12 consecutive months prior to the first dosing and surgically sterile females who have undergone hysterectomy, bilateral oophorectomy, or tubal ligation at least 6 months prior to the first dosing) must be willing to use one of the following acceptable contraceptive methods throughout the study and for 90 days after the last dosing:

   a. simultaneous use of a male condom and, for the female partner, hormonal contraceptives or intra-uterine contraceptive device used since at least 4 weeks prior to the first dosing;

   b. simultaneous use of a male condom and, for the female partner, a diaphragm or cervical cap with intravaginally applied spermicide.

8. Male subjects (including men who have had a vasectomy) with a pregnant partner must agree to use a condom from the first dosing and for 90 days after the last dosing.

9. Male subjects must be willing not to donate sperm for at least 90 days after the last dosing.

10. Subjects must be able to understand the nature of the study, agree to comply with the prescribed dosage regimens, and communicate to study personnel about AEs and concomitant medication use, as applicable.

11. Subjects must sign and date the IEC-approved informed consent obtained prior to the conduct of any study activities.

12. Subjects must be willing to remove dentures or mouth piercing, if applicable, at the time of dosing.

8.3 Exclusion Criteria

Subjects to whom any of the following applies will be excluded from the study:

Medical History and Concurrent Diseases

1. Current diagnosis of viral hepatitis or a history of liver disease.

2. Significant history of seizure disorder other than a single childhood febrile seizure (e.g., epilepsy).

3. Current or recent (within 3 months of study drug administration) clinically significant gastrointestinal disease that may interfere with drug absorption.

4. Gastrointestinal surgery that interferes with physiological absorption and motility (i.e., gastric bypass, duodenectomy, or gastric banding).

5. History of significant alcohol abuse or regular use of alcohol within 6 months prior to screening (more than 14 units for males or 7 units for females of alcohol per week [1 unit = 150 mL of wine, 360 mL of beer, or 45 mL of 40% alcohol]).

6. History of significant drug abuse within 6 months prior to screening or use of soft drugs (such as marijuana) within 3 months or hard drugs (such as cocaine, PCP, crack, opioid derivatives including heroin, and amphetamine derivatives) within 1 year prior to screening.

7. History of allergic reactions to rimegepant, cyclosporine, quinidine, or other related drugs, or to any excipient in the formulations.

8. History of anaphylaxis, documented hypersensitivity reaction, or clinically significant reaction to any drug.

9. Clinically significant history of depression or suicidal thoughts within 6 months prior to screening.

10. Use of medications for the timeframes specified below, with the exception of medications exempted by the Investigator on a case-by-case basis if they are judged unlikely to affect the PK profile of the study drug or subject safety (e.g., topical drug products without significant systemic absorption):

    a. prescription medications within 14 days prior to the first dosing;

    b. over-the-counter products (including acetaminophen-containing products) and natural health products (including herbal remedies, homeopathic and traditional medicines, probiotics, food supplements such as vitamins, minerals, amino acids, essential fatty acids, and protein supplements used in sports) within 14 days prior to the first dosing, with the exception of the occasional use of ibuprofen;

    c. live attenuated vaccine within 1 month prior to the first dosing or planned vaccination during the course of the study;

    d. injectable or implantable hormonal contraceptive agent within 2 months prior to the first dosing;

    e. depot injection or implant of any other drug within 3 months prior to the first dosing;

    f. any drug known to induce or inhibit hepatic drug metabolism, including St. John's wort, within 1 month prior to the first dosing.

11. Donation of plasma within 7 days prior to dosing. Donation or loss of blood (excluding volume drawn at screening) of 50 mL to 499 mL of blood within 30 days, or more than 499 mL within 56 days prior to the first dosing.

12. Participation in a clinical research study involving the administration of an investigational or marketed drug or device within 30 days prior to the first dosing, administration of a biological product in the context of a clinical research study within 90 days prior to the first dosing, or concomitant participation in an investigational study involving no drug or device administration.

13. For subjects participating in Part I of the study:

    a. History of latent or active tuberculosis or exposure to endemic areas within 8 weeks prior to QuantiFERON®-TB testing performed at screening.

    b. Positive QuantiFERON®-TB test indicating possible tuberculosis infection.

    c. History of clinically significant opportunistic infection (e.g., invasive candidiasis or pneumocystis pneumonia).

    d. Serious local infection (e.g., cellulitis, abscess) or systemic infection (e.g., septicemia) within 3 months prior to screening.

    e. Clinically significant history of uncontrolled hypertension.

    f. History of active malignancy.

    g. History of psoriasis, except if mild psoriasis (small number of minor plaques requiring no treatment or only intermittent topical steroids).

14. For subjects participating in Part II of the study:

    a. History of myasthenia gravis, optic neuritis, prolongation of the QT interval, ventricular arrhythmias, thrombocytopenia, atrial fibrillation or flutter, uncorrected hypokalemia, or bradycardia.

    b. Have previously been dosed in Part I of the study.

Physical and Laboratory Test Findings

15. Any abnormal laboratory test results deemed clinically significant by the Investigator or positive test during medical screening.

16. Positive test for HIV, HBsAg, or HCV during medical screening.

17. Evidence of organ dysfunction or any clinically significant deviation from normal on physical examination, vital signs, 12-lead ECG, or clinical laboratory determinations beyond what is consistent with the target population.

18. Any of the following laboratory parameters above the ULN values at screening or Day -1: ALP, AST, ALT, GGT, direct bilirubin, indirect bilirubin, and total bilirubin. Only abnormal values between 1-1.5x ULN may be repeated once for confirmation to below ULN.

19. Any of the following abnormalities on 12-lead ECG or BP at screening, confirmed by repeat:
    a. PR ≥210 msec.
    b. QRS ≥120 msec.
    c. QTcF >450 msec.
    d. Sitting (for at least 5 minutes) systolic BP >140 mmHg, confirmed by repeat.
    e. Sitting (for at least 5 minutes) diastolic BP >90 mmHg, confirmed by repeat.
20. Any of the following abnormal laboratory test values at screening or Day -1:
    a. Hemoglobin <128 g/L for males and <115 g/L for females.
    b. Hematocrit <0.37 L/L for males and <0.32 L/L for females.
    c. Total WBC <3.0 x 10^9/L.
    d. Platelet count <100 x 10^9/L.
    e. Neutrophils <1.4 x 10^9/L and <1.0 x 10^9/L for Afro-American volunteers.
    f. CPK >2x ULN.
21. Positive urine drug screen, urine cotinine test, or alcohol breath test at screening or Day -1.
22. Presence of fever (body temperature >37.6°C) (e.g., a fever associated with a symptomatic viral or bacterial infection) within 2 weeks prior to the first dosing.
23. Presence of orthodontic braces or orthodontic retention wires, or any physical finding in the mouth or tongue that would be likely to interfere with successful completion of the dosing procedure.
24. Inability to be venipunctured and/or tolerate catheter venous access.
25. Inability or difficulty swallowing tablets or capsules.
26. Any reason which, in the opinion of the Investigator, would prevent the subject from participating in the study.

9 CLINICAL PROCEDURES

Unless otherwise specified, procedures, data collection and evaluation will be conducted as per the clinical site SOPs.

9.1 Screening Procedures

Subject screening procedures will be performed within 28 days preceding administration of study medication. Subjects must provide written informed consent prior to initiation of any screening procedures. The consent to perform some general screening procedures may be obtained on a consent document other than the Informed Consent Form (ICF) specific to this study, and therefore, some screening test results could be obtained before signature of the ICF specific to this study. The study-specific ICF must be signed and dated by the subject before participation to study-specific procedures.

Screening procedures will include: Demographic data, medical and medication histories, complete physical examination, body measurements, S-STS, vital signs (BP, HR, RR, and OT), 12-lead ECG, hematology, biochemistry, serology (HIV, HBsAg, and HCV), urinalysis, urine pregnancy test, urine drug screen, urine cotinine test, and alcohol breath test.

For subjects in Part I, a QuantiFERON®-TB test will also be performed, unless documented results are available within 2 months preceding dosing. In any case, the standard tuberculosis medical history screening questionnaire will be administered to each subject.

For eligibility purposes, abnormal laboratory or vital signs results may be repeated once if abnormal result is observed at the initial reading. Moreover, abnormalities found in the ECG may need to be confirmed by repeated measurements. In the event that the participation of a subject in the study is delayed and some screening procedures had been performed outside of the prescribed screening window, outdated screening procedures can be repeated.

9.2 Confinements, Visits and Washout

For each period, subjects will be confined from the morning of Day -1 until after the 24-hour post-dose blood draw on Day 2. Subjects will come back for all subsequent blood draws.

For Part I, there will be a washout period of at least 14 days between doses. Participation of each subject should last approximately 3 weeks.

For Part II, there will be a washout period of at least 7 days between doses. Participation of each subject should last approximately 2 weeks.

9.3 Randomization and Blinding

This study will be open-label in nature. Subjects will be administered each treatment according to the two-period, two-sequence, block randomization scheme produced for each study part separately. The randomization code will not be available to the Bioanalytical Division of Syneos Health until the clinical and analytical phases of each study part have been completed.

9.4 Study Treatments

Part I:

In each period, subjects will receive one of the following treatments:

Treatment A: 1 x 75 mg rimegepant ODT (Nurtec ODT; Biohaven Pharmaceuticals, Inc., USA) to be held under the tongue (sublingual administration) until fully dissolved then swallowed without water, administered under fasting conditions.

Treatment B: 1 x 75 mg rimegepant ODT co-administered with 2 x 100 mg cyclosporine capsules (Neoral; Novartis Pharmaceuticals Canada Inc., Canada) under fasting conditions.

Part II:

In each period, subjects will receive one of the following treatments:

Treatment A: 1 x 75 mg rimegepant ODT (Nurtec ODT; Biohaven Pharmaceuticals, Inc., USA) to be held under the tongue (sublingual administration) until fully dissolved then swallowed without water, administered under fasting conditions.

Treatment C: 1 x 75 mg rimegepant ODT co-administered with 2 x 300 mg quinidine (as sulfate) tablets (Quinidine Sulfate Tablets; Sandoz Inc., USA) under fasting conditions.

9.5 Drug Supplies and Accountability

It is the responsibility of the Sponsor to ensure that study medication provided for this study are manufactured under Good Manufacturing Practice (GMP) and are suitable for human use. The Sponsor is responsible to ship a sufficient amount of dosage units to allow the clinical site to maintain an appropriate sampling for the study. The study drugs will be stored at the clinical site as per applicable requirements.

Labels will be provided in appropriate languages as required by the country in which the study is conducted. The content of the labeling will be in accordance with local regulatory specifications and requirements. The study drugs will be stored in a locked, environmentally-controlled medication room with restricted access. Container(s) will bear a label containing at least the name of the study drug, lot and/or batch number, and manufacturing and/or expiry/retest date. Individual subject doses will be dispensed according to the randomization scheme in appropriate envelopes/containers indicated with at least the project number, the period number, and the subject number/spare number. The study drugs will be dispensed according to the clinical site SOP.

The study drugs received at the site will be inventoried and accounted for throughout the study and the result recorded in the drug accountability/retention record according to the clinical site appropriate SOP. Upon completion of the study, the remaining drug products will be maintained at the clinical site, discarded, or returned to the Sponsor, as per Sponsor's request.

9.6 Drug Administration

For all treatments, one rimegepant ODT will be placed under each subject's tongue by the clinical staff; time of dosing ("0") will be set as the time the ODT is placed under the tongue. Subjects will be instructed not to swallow saliva until the ODT is completely dissolved. Subjects will be instructed to give a hand sign once the ODT is completely dissolved and swallowed. A hand and mouth check will be performed to ensure consumption of the medication. If the ODT is not completely dissolved within 2 minutes, subjects will be asked to swallow the remaining with saliva and this will be documented. The start and end time of complete dosing procedure will be recorded. Subjects will be required to remove dentures or mouth piercing at the time of dosing.

For Treatment B, two cyclosporine capsules will be administered with approximately 240 mL of water within 2 minutes prior to rimegepant ODT administration. The capsules must be swallowed whole and subjects will be advised to not crush or chew the medication. A hand and mouth check will be performed to ensure consumption of the medication. The time of administration of both drugs will be recorded, and the time interval between cyclosporine and rimegepant administrations should not exceed 2 minutes.

For Treatment C, two quinidine tablets will be administered with approximately 240 mL of water within 2 minutes prior to rimegepant ODT administration. The tablets must be swallowed whole and subjects will be advised to not crush or chew the medication. A hand and mouth check will be performed to ensure consumption of the medication. The time of administration of both drugs will be recorded, and the time interval between quinidine and rimegepant administrations should not exceed 2 minutes.

9.7 Study Restrictions

9.7.1 Food and Fluids

No food will be allowed from at least 10 hours before dosing until at least 4 hours post-dose. Meals will be standardized and similar in composition between periods.

Except for water given for Treatments B and C, no fluids will be allowed from 1 hour before dosing until 1 hour post-dose. Water will be provided *ad libitum* at all other times.

In addition, subjects will be required to abstain from:

- Food or beverages containing grapefruit, starfruit, pomegranate, pineapple, or pomelo from 14 days prior to the first dose until after the last PK blood sample collection of the study;
- Food or beverages containing xanthine derivatives or xanthine-related compounds or energy drinks from 48 hours prior to dosing until after the last PK blood sample collection of each period;

- Food containing poppy seeds within 24 hours prior to admission of each period.

9.7.2 Tobacco, Alcohol and Illicit Drugs

Subjects will be required to abstain from using soft or hard drugs or any tobacco or nicotine products from screening and throughout the study.

Consumption of alcohol-based products will be prohibited from 24 hours prior to admission until after the last PK blood sample collection of each period.

9.7.3 Concomitant Medications

Subjects will be required to avoid using prescription medications, over-the-counter products (including acetaminophen-containing products), and natural health products (including herbal remedies, homeopathic and traditional medicines, probiotics, food supplements such as vitamins, minerals, amino acids, essential fatty acids, and protein supplements used in sports) for the period of time specified in exclusion criterion no.10 and throughout the study.

No concomitant medications are allowed during the study, with the exception of one(s) required for the medical management of an AE, medications exempted by the Investigator on a case-by-case basis that are judged unlikely to affect the PK profile of the study drug or subject safety (e.g., topical drug products without significant systemic absorption) and occasional use of ibuprofen.

All medications taken by subjects after screening until the last study day will be documented as concomitant medications. Any concomitant medication use, other than the allowed medications stated above, will be reviewed and evaluated on a case-by-case basis by the Investigator to determine if they affect a subject’s eligibility or continued participation in the study, or for potential impact on the study results.

Subjects will be notified not to receive any live attenuated vaccine from 1 month prior to the first dosing and during the study.

9.7.4 Posture and Physical Activity

For safety reasons, subjects will be required to remain seated and avoid lying down or sleeping for the first 4 hours after drug administration. However, failure of subjects to comply with these requirements does not constitute a deviation from the protocol if it is medically necessary, required for procedures, or to go to the bathroom. When appropriate, subjects will be accompanied by a staff member while walking.

Because excessive physical activity may increase the level of CPK above the ULN value, subjects will be advised to avoid performing such activity at all times during the study duration and especially for 3 days before each study drug administration and 3 days before study exit procedures. Vigorous activity will be prohibited at all times during the confinement.

9.7.5 Other Restrictions

Subjects will be advised not to travel to countries where tuberculosis is endemic during the study and for 14 days after the last dosing.

Subjects will be advised to avoid close contact with people with bacterial or viral infections (e.g., flu, gastroenteritis, etc), during the study.

9.8 Sample Collection and Processing

In each period, a total of 20 blood samples will be drawn from each subject for PK analyses. Blood samples will be collected prior to drug administration and 0.083, 0.167, 0.333, 0.5, 0.667, 0.833, 1, 1.5, 2, 2.5, 3, 4, 5, 6, 8, 12, 24 (Day 2), 48 (Day 3), and 72 (Day 4) hours post-dose (3 mL for each sampling time).

The time tolerance window for blood samples collected during the confinement period will be ±1 minute for all samples collected before 8 hours post-dose and ±3 minutes for subsequent samples. The time tolerance window for return visit samples will be ±30 minutes. Sample collections done outside the pre-defined time windows will not be considered as protocol deviations since actual post-dose sampling times will be used for PK and statistical analyses. Unless otherwise specified or for subject safety, when blood draws and other procedures coincide, blood draws will have precedence. A dead-volume IV catheter will be used for blood collection to avoid multiple skin punctures, when appropriate. Otherwise, blood samples will be collected by direct venipuncture.

The total volume of blood including that collected for eligibility and safety purposes should not exceed 195 mL for the whole study.

Plasma samples will be collected and processed as per the Analytical Methodology Information Sheet.

9.9 Safety Monitoring

Subjects will be monitored throughout the study by the clinical staff for AEs. In each period, a physician will be on site for drug administration and until 4 hours post-dose, and available on call for the remainder of the study. If necessary, a physician, either at the clinical site or in a nearby hospital will administer treatment for any AE. A crash cart or emergency bag containing the necessary rescue material and appropriate medications will be available in the clinic to allow rapid intervention in case of emergency.

Safety parameters, including laboratory results and ECG, will be assessed by a physician, using the clinical site's criteria for biomedical laboratory and ECG acceptance ranges as suggested guidelines in making the medical assessment.

Scheduled safety measurements will be repeated according to the clinical site SOPs or upon request from a physician. Any abnormal repeated measurement will be evaluated by a physician and repeated if judged necessary. Further action may be taken upon physician's request.

Subjects will be advised to notify their health care professional(s) (e.g., physician, dentist, and/or pharmacist) that they are participating in a clinical research study on a drug called rimegepant co-administered with cyclosporine or quinidine before taking any medicines or undergoing any medical procedure.

9.9.1 Physical Examination

A complete physical examination will be performed at screening. A complete physical examination includes assessments of the following: head, eyes, ears, nose, throat (HEENT), neck, chest, lungs, abdomen, musculoskeletal, dermatological, cardiovascular/peripheral vascular, and general neurological examination.

A brief physical examination will be performed on Day -1 in each period and at study exit. A brief physical examination includes assessments of the following: HEENT, chest, lungs, abdomen, dermatological, cardiovascular/peripheral vascular, and areas of note elicited from the subject.

9.9.2 Body Measurements

Body measurements will be performed at screening and will include body weight, height measurement, and BMI calculation.

9.9.3 Sheehan Suicidality Tracking Scale

S-STS will be performed at screening and at study exit. The S-STS is a prospective, patient self-reported or clinically administered rating scale that contains 16 questions to track both treatment-emergent suicidal ideation and behaviors.[13,14]

This scale will be administered by a member of the medical team, completed on site, and will be in paper. The source document will be provided by the Sponsor. The assessment period for completing the scale will be 6 months prior to screening for the assessment at the screening visit and since screening for the assessment at the study exit visit.

If the Investigator determines that a subject is at risk of suicide or self-harm, appropriate measures to ensure the subject's safety and obtain mental health evaluation must be implemented. The subject must immediately be discontinued from the study. The event should be recorded as either an AE or a SAE as determined by the Investigator and reported within 24 hours to the Sponsor.

9.9.4 Vital Signs

BP, HR, RR, and OT will be measured in a sitting position (except for safety reasons) at screening and at study exit. OT will also be measured on Day -1, before dosing on Day 1, and approximately 24, 48, and 72 hours post-dose in each period.

For Part I, BP and HR will be measured before dosing on Day 1 and approximately 2, 4, 6, 24, 48, and 72 hours post-dose in each period.

For Part II, BP and HR will be measured before dosing on Day 1 and approximately 1, 2, 4, 6, 8, 12, 24, 48, and 72 hours post-dose in each period.

When vital signs measurements coincide with a blood draw, they should preferably be performed before the blood collection whenever possible. Vital signs performed 72 hours post-dose of Period 2 can be used as the vital signs required at study exit.

9.9.5 ECG

Supine 12-lead-ECG will be performed at screening and at study exit.

For Part I, supine 12-lead ECG will be performed before dosing on Day 1 in each period.

For Part II, supine 12-lead ECG will be performed approximately 24 hours post-dose in each period.

When ECG coincides with a blood draw, it should preferably be performed before the blood collection whenever possible.

9.9.6 Cardiac Telemetry

For Part II, continuous cardiac monitoring will be performed by telemetry, in accordance with the clinical site SOP, from approximately 10 hours pre-dose until 6 hours post-dose in each period, and will include real time measurements of heart rate and cardiac rhythm. 12-lead ECG will be extracted before dosing on Day 1 and approximately 1, 2, 4, and 6 hours post-dose in each period.

9.9.7 Laboratory Assessments

9.9.7.1 QuantiFERON®-TB Test

For subjects in Part I, a QuantiFERON®-TB test will be performed at screening. This test will be performed unless documented results are available within 2 months preceding dosing. In any case, the standard tuberculosis medical history screening questionnaire will be administered to each subject.

9.9.7.2 Pregnancy Test

A urine pregnancy test will be performed at screening and at study exit, and a serum pregnancy test will be performed on Day -1 in each period.

9.9.7.3 Drug, Alcohol and Cotinine *Screen*

A urine drug screen (amphetamines, methamphetamines, barbiturates, benzodiazepines, tetrahydrocannabinol, cocaine, opiates, PCP, methadone), a urine cotinine test, and an alcohol breath test will be performed at screening and on Day -1 in each period.

9.9.7.4 Hematology

Hematology will be performed at screening, on Day -1 in each period, and at study exit. The following will be assessed: complete blood count with differential, hemoglobin, and hematocrit.

Haptoglobin and reticulocyte count will be performed in case of decrease in hemoglobin levels below the LLN on Day -1 in each period and at study exit.

If the haptoglobin and reticulocyte count results come back normal but the hemoglobin levels results are still abnormal after the repeat, the haptoglobin and reticulocyte count tests may be repeated upon Investigator's judgement.

9.9.7.5 Biochemistry

Biochemistry will be performed at screening, on Day -1 in each period, and at study exit. The following will be assessed: albumin, ALP, AST, ALT, GGT, urea, calcium, chloride, glucose, phosphorus, potassium, creatinine, sodium, CPK, direct bilirubin, indirect bilirubin, total bilirubin, and total protein.

Considering that indirect bilirubin is calculated from total and direct bilirubin values, indirect bilirubin result would not be available in case of direct bilirubin below the limit of quantification.

9.9.7.6 Coagulation

Coagulation tests will be performed in case of abnormal LFT (i.e., ALP, AST, or ALT is >3x ULN) on Day -1 in each period and at study exit. The following will be assessed: prothrombin time (PT)/international normalized ratio (INR) and activated partial thromboplastin time (aPTT).

If the coagulation tests results come back normal but the LFT results are still abnormal after the repeat, the coagulation tests may be repeated upon Investigator's judgement.

9.9.7.7 Serology

HIV antigen and antibody, HBsAg, and HCV antibody will be performed at screening.

9.9.7.8 Urinalysis

Urinalysis will be performed at screening, on Day -1 in each period, and at study exit. The following will be assessed: macroscopic examination, pH, specific gravity, protein, glucose, ketones, bilirubin, occult blood, nitrite, urobilinogen, and leukocytes. Unless otherwise specified, microscopic examination will be performed on abnormal findings.

9.10 Study Exit/Early Termination Procedures

Study exit/early termination procedures will include: Brief physical examination, S-STS, vital signs (BP, HR, RR, and OT), 12-lead ECG, hematology, (including haptoglobin and reticulocyte count in case of decrease in hemoglobin levels below the LLN), biochemistry (including

coagulation in case of abnormal LFT), urinalysis, urine pregnancy test, concomitant medications, and AE monitoring.

Study exit procedures are scheduled to be performed at the last study day. If not possible, or in case of early termination, all efforts will be made to complete study exit/early termination procedures within 14 days after the last participation of the subject in the study.

9.11 Data Collection and Evaluation

Subjects' personal information will be stored in an electronic data capture (EDC) system (Initiator™ or Alphadas®). All clinical raw data will be recorded promptly, accurately, and legibly; either directly into the EDC system as e-source data or indelibly on paper (e.g., raw data sheets when electronic data capture is not possible). A detailed list of the type (electronic or paper) and location for all source data will be included in the Trial Master File. When recorded electronically, Case Report Forms will be electronically generated afterwards. All raw data will be conserved in order to maintain data integrity. The Investigator and/or the clinical staff have the responsibility of ensuring the completeness and accuracy of the clinical data.

All laboratory results provided by Biron biomedical laboratory will be stored in InLab (Clinical Laboratory Information Management System). Initiator™, Alphadas®, and InLab are validated and are Code of Federal Regulations (CFR) part 11 compliant applications.

9.12 Subject Withdrawal and Replacement

Subjects will be advised that they are free to withdraw from the study at any time. Over the course of the study, the Sponsor and the Investigator or a delegate may withdraw any subject from the study for one of the reasons described below; subject withdrawal will be done in accordance with the clinical site's SOP:

- Safety reason;
- Non-compliance with protocol requirements;
- Significant protocol deviation;
- Positive pregnancy test, urine drug screen, urine cotinine test, or alcohol breath test;
- Vomiting within 3 hours after dosing for Treatment A and Treatment B or within 4 hours after dosing for Treatment C.

Clinical laboratory results will be reviewed by the Investigator and subjects will be withdrawn from the study if it is deemed that the subject's safety may be at risk on the basis of these test results.

Subjects who withdraw or are withdrawn from the study after dosing will not be replaced. However, in the event that the number of drop-outs exceeds initial expectations, subjects who withdraw or are withdrawn might be replaced at the discretion of the Sponsor. Such replacement

resulting in dosing more subjects than planned in this protocol would be documented in a protocol amendment.

Subjects who withdraw or are withdrawn will be asked to remain at the clinic until the Investigator or a delegate agrees that the subject is fine and can be discharged. As soon as subject withdrawal is confirmed, blood sampling will be stopped. A PK blood draw may be collected at the time of withdrawal if deemed required by the Investigator. Study exit procedures will be performed at the time of withdrawal from the study or as soon as possible thereafter.

9.13 Adverse Events

9.13.1 Recording of Adverse Events

AEs, including any alteration in the oral cavity, will be recorded and evaluated for their seriousness, severity, and relationship to the study medication. AEs will be collected and documented during the course of the study after signature of the ICF. For a period of 4 days following the last drug administration, AEs will also be documented if reported. AEs will be followed-up until complete resolution, or until the Investigator or Medical Sub-Investigator judges safe to discontinue follow-up. The relationship to the study medication will be classified according to section 9.13.3.

9.13.2 Assessment of Severity

The severity of AEs will be described and documented using the following definitions:

**Severity**	**Description**
**Mild**	Awareness of signs and symptoms, but are easily tolerated; are of minor irritant type; causing no limitations of usual activities. Signs or symptoms may require minor action.
**Moderate**	Discomfort severe enough to cause some limitations of usual activities and may require action.
**Severe**	Incapacitating with inability to carry out usual activities or significantly affects clinical status, and requires specific action and/or medical attention.

9.13.3 Assessment for Determining Relationship to Study Drug

Each AE must be classified based on medical judgment and according to the following categories: Probably related, Possibly related, Remotely related, and Unrelated (not related).

The definitions for the causality assessments are as follows:

- **PROBABLY RELATED** (must have first three points)**:** This category applies to AEs that are considered, with a high degree of certainty, to be related to the investigational product. An AE may be considered probable, if:

1. It follows a reasonable temporal sequence from the administration of the drug.

2. It cannot be reasonably explained by the known characteristics of the subject's clinical state, environmental or toxic factors or other modes of therapy administered to the subject.
3. It disappears or decreases on cessation or reduction in dose (there are important exceptions when an AE does not disappear upon discontinuation of the drug, yet drug relatedness clearly exists; e.g., (1) bone marrow depression, (2) tardive dyskinesias).
4. It follows a known pattern of response to the suspected drug.
5. It reappears upon re-challenge.

- **POSSIBLY RELATED** (must have first two points)**:** This category applies to AEs in which the connection with the investigational product administration appears unlikely but cannot be ruled out with certainty. An AE may be considered possible if, or when:
    1. It follows a reasonable temporal sequence from the administration of the drug.
    2. It may have been produced by the subject's clinical state, environmental or toxic factors, or other modes of therapy administered to the subject.
    3. It follows a known pattern of response to the suspected drug.
- **REMOTELY RELATED** (must have first two points)**:** In general, this category is applicable to an AE that meets the following criteria:
    1. It does not follow a reasonable temporal sequence from the administration of the investigational product.
    2. It may readily have been produced by the subject's clinical state, environmental or toxic factors, or other modes of therapy administered to the subject.
    3. It does not follow a known pattern of response to the suspected drug.
    4. It does not reappear or worsen when the investigational product is re-administered.
- **UNRELATED (NOT RELATED):** This category is applicable to AEs that are judged to be clearly and incontrovertibly due only to extraneous causes (disease, environment, etc.), and do not meet the criteria for medication relationship listed under remote, possible, or probable.

Determination of the relationship of AEs to the study drug:

	Probably related	Possibly related	Remotely related	Unrelated (not related)
Clearly due to extraneous causes	–	–	–	+
Reasonable temporal association with drug administration	+	+	–	–
May be produced by subject clinical state, etc.	–	+	+	+
Known response pattern to suspected drug	+	+	–	–
Disappears or decreases on cessation or reduction of the dose	+	–	–	–
Reappears on re-challenge	+	–	–	–

9.13.4 Serious Adverse Events

9.13.4.1 Definition of Serious Adverse Event

A SAE is any event that meets any of the following criteria:

- Death
- Life-threatening
- Inpatient hospitalization or prolongation of existing hospitalization
- Persistent or significant disability/incapacity
- Congenital anomaly/birth defect in the offspring of a subject
- Other: Important medical events that may not result in death, be life-threatening, or require hospitalization, may be considered an SAE when, based upon appropriate medical judgment, they may jeopardize the subject and may require medical or surgical intervention to prevent one of the outcomes listed in this definition. Examples of such events are:
    - Intensive treatment in an emergency room or at home for allergic bronchospasm
    - Blood dyscrasias or convulsions that do not result in inpatient hospitalization
    - Development of drug dependency or drug abuse

9.13.4.2 Definition of Terms

Life-threatening: An AE is life-threatening if the subject was at immediate risk of death from the event as it occurred; i.e., it does not include a reaction that if it had occurred in a more serious form might have caused death. For example, drug induced hepatitis that resolved without evidence of hepatic failure would not be considered life threatening even though drug induced hepatitis can be fatal.

Hospitalization: AEs requiring hospitalization should be considered SAEs. Hospitalization for elective surgery or routine clinical procedures that are not the result of AE (e.g., elective surgery for a pre-existing condition that has not worsened) need not be considered AEs or SAEs. If anything untoward is reported during the procedure, that occurrence must be reported as an AE, either 'serious' or 'non-serious' according to the usual criteria.

In general, hospitalization signifies that the subject has been detained (usually involving at least an overnight stay) at the hospital or emergency ward for observation and/or treatment that would not have been appropriate in the physician's office or outpatient setting. When in doubt as to whether 'hospitalization' occurred or was necessary, the AE should be considered serious.

Disability/incapacitating: An AE is incapacitating or disabling if the experience results in a substantial and/or permanent disruption of the subject's ability to carry out normal life functions.

Any SAE will be reported to the Sponsor via telephone, fax, e-mail or in person, within 24 hours of knowledge by the Investigator, and then in writing as soon as possible, but no later than 7 calendar days after first knowledge of the SAE.

The notification must be directed to:

9.13.5 Suspected, Unexpected, Serious Adverse Drug Reactions

9.13.5.1 Fatal or Life-threatening Serious, Unexpected Adverse Drug Reactions

The Sponsor is responsible for notifying regulatory agencies of fatal or life-threatening serious, unexpected adverse drug reactions (by telephone, facsimile transmission or in writing) as soon as possible, but no later than 7 calendar days after becoming aware of the information. Additionally, within 8 days after having informed the agency(ies), a complete report must be submitted, including an assessment of the importance and implication of any findings. Syneos Health will handle notifications to the Canadian regulatory agency on behalf of the Sponsor.

It is the responsibility of the clinical site to report as soon as possible, but no later than 7 calendar days after first knowledge by the Investigator, fatal or life-threatening serious, unexpected adverse drug reactions to the IEC responsible for the study.

9.13.5.2 Other Suspected, Unexpected, Serious Adverse Drug Reactions

The Sponsor is responsible for notifying regulatory agencies of all other suspected, unexpected, serious adverse drug reactions that are neither fatal nor life-threatening as soon as possible, but no later than 15 calendar days after becoming aware of the information. Syneos Health will handle notifications to the Canadian regulatory agency on behalf of the Sponsor.

It is the responsibility of the clinical site to report to the IEC responsible for the study all other suspected, unexpected, serious adverse drug reactions that are neither fatal nor life-threatening, as soon as possible, but no later than 15 calendar days after first knowledge by the Investigator.

9.14 Pregnancy

If a subject or partner of a subject participating in the study becomes pregnant during the study, the Investigator should report the pregnancy to the Sponsor within 24 hours of being notified.

A subject becoming pregnant while on study drug will immediately be withdrawn from the study and early termination study procedures will be performed.

The subject or partner should be followed by the Investigator (after obtaining the consent of the female partner, when applicable) until completion of the pregnancy. If the pregnancy ends for any reason before the anticipated date, the Investigator should notify the Sponsor. At the completion of the pregnancy, the Investigator will document the outcome of the pregnancy.

If the outcome of the pregnancy meets the criteria for immediate classification as a SAE (i.e., postpartum complication, spontaneous abortion, stillbirth, neonatal death, or congenital anomaly), the Investigator should follow the procedures for reporting an SAE.

9.15 Reportable Disease

In the case a subject has or manifests any clinical signs characteristic of a reportable disease or condition (e.g., HIV, tuberculosis, SARS), it is the responsibility of the Medical Director to notify the Public Health authorities within 48 hours after becoming aware of the information.

9.16 Premature Termination of the Study

The study may be prematurely terminated by the Investigator following consultation with the Sponsor, by the Sponsor or by the regulatory authorities. Following a decision to discontinue the trial, the Investigator will promptly inform the active study subjects and the IEC responsible for this trial, stating the reasons for discontinuation of the study and, furthermore, advise them in writing of any potential risks to the health of study subjects or other persons. It is the Sponsor's responsibility to report the premature termination of the study to the regulatory authority(ies), when required by the applicable regulatory requirement(s). The Canadian regulatory agency must be informed of premature termination within 15 days, provided with the reasons for the trial discontinuation and of any potential risks to the health of study subjects or other persons. Syneos Health may notify the Canadian regulatory agency on behalf of the Sponsor upon his request.

10 ANALYTICAL METHODOLOGY

When applicable, samples will be transported to the bioanalytical facility in at least two separate shipments, with each set of aliquots in separate shipments. Once the bioanalytical laboratory confirms receipt of the first shipment, the second set of aliquots may be sent. The samples should be packed on sufficient dry ice to keep them frozen for at least 72 hours.

The Bioanalytical Division of Syneos Health will analyze rimegepant in plasma samples using a validated method.

Analyst and Watson LIMS (Laboratory Information Management System) will be used at different steps of the analysis.

The bioanalytical work in support of the study will be conducted in compliance with the GCP, using the SOPs in place in the Bioanalytical Division of Syneos Health. These SOPs are in accordance with applicable regulations in the industry: Guidelines on Bioanalytical Method Validation, Good Laboratory Practice (GLP), and Guideline for GCP ICH E6 (R2).

Samples from subjects included in the PK population (see section 11.2.1) and from subjects who were withdrawn from the study due to adverse events or vomiting episodes will be analyzed.

11 PHARMACOKINETIC AND STATISTICAL ANALYSES

PK analyses will be performed using Phoenix® WinNonlin®. Inferential statistical analyses will be performed using SAS® according to FDA guidelines.

For each study part, bioanalysis of samples should be completed prior to the initiation of the PK and statistical analyses.

11.1 Pharmacokinetic Parameters

The following PK parameters will be calculated by standard non-compartmental methods for rimegepant:

1. AUC_{0-t}: area under the concentration-time curve from time zero until the last observed concentration

2. AUC_{0-inf}: area under the concentration-time curve from time zero to infinity (extrapolated)

3. C_{max}: maximal observed concentration

4. Residual area: calculated as $(1 – (AUC_{0-t} / AUC_{0-inf})) \times 100$

5. T_{max}: time when the maximal concentration is observed

6. $T_{½ el}$: terminal elimination half-life

7. K_{el}: terminal elimination rate constant

Additional PK analysis may be performed. Upon Sponsor's request, PK repeats might be performed according to Syneos Health's SOP. If re-assays are requested for PK reasons, final results will include re-assay values, while results with original values will be presented in an appendix of the clinical study report as supportive data.

11.2 Analysis Populations

11.2.1 Pharmacokinetic Population

The PK population will include all subjects completing Part I or Part II of the study and for whom the PK profile can be adequately characterized.

Any subject with pre-dose concentrations will be presented in the concentrations and PK tables but excluded from descriptive statistics and ANOVA if the pre-dose concentration is greater than 5% of the C_{max} value of that period for that subject.

Data from subjects who experienced emesis during the sampling interval and who were not withdrawn as per criteria established under section 9.12 may be evaluated after completion of the PK analyses. Any subject who experienced emesis within 2 times the median T_{max} obtained for the specific treatment received in the current study will be excluded from the statistical analyses (i.e., descriptive statistics and ANOVA). Subjects withdrawn due to AEs will be presented in the data listings but excluded from the statistical analysis tables.

11.2.2 Safety Population

The safety population is defined as all subjects who received at least one dose of the study medication.

11.3 Statistical Analyses

Details of statistical analyses will be developed in a SAP. The SAP will be prepared after completion of the final protocol and finalized prior to database lock.

11.3.1 Pharmacokinetic Analyses

Individual and mean plasma concentration versus time curves will be presented for both linear and semi-log scales. Descriptive statistics (arithmetic and geometric means, standard deviation [SD], coefficient of variation [CV%], minimum [Min], maximum [Max], and median) of the plasma concentrations and the PK parameters will be presented.

Using GLM procedures in SAS, ANOVA will be performed on untransformed T_{max}, K_{el}, and $T_{½ el}$ and on ln-transformed AUC_{0-t}, AUC_{0-inf}, and C_{max} at the alpha level of 0.05. The model will include treatment, sequence, and period as fixed effects, and subject nested within sequence as a random effect. If the study doses in more than one group, the statistical model will be modified to reflect the multigroup nature of the study. In the case of a non-statistically significant treatment-by-group interaction term, the analysis will be rerun excluding this term from the ANOVA model in order to obtain ratios and CI where appropriate. Intra- and inter-subject CV will be estimated. The ratio of geometric means (B/A for Part I and C/A for Part II) and 90% CI for the ratio of geometric means, based on least-squares means from the ANOVA of the ln-transformed data, will be calculated for AUC_{0-t}, AUC_{0-inf}, and C_{max}.

The 90% CI for the ratios of AUC_{0-inf} and C_{max} will be used to quantify the extent of drug interaction.

The statistical analyses will be performed separately for each study part. Interim PK analysis and review of data will be performed following Part I completion. Part II will be conducted if rimegepant AUC_{0-inf} ratio of geometric means in Part I is increased by more than 50% when co-administered with cyclosporine.

Additional statistical analysis may be performed.

11.3.2 Safety and Tolerability Analyses

Demographic parameters will be summarized descriptively.

Safety and tolerability will be evaluated through the assessment of AEs (i.e., seriousness, severity, relationship to the study medication, outcome, duration, and management), vital signs, 12-lead ECG, clinical laboratory parameters and physical examination. TEAEs will be tabulated by treatment. AEs will be coded using the latest version of the MedDRA. Safety and tolerability data will be reported using descriptive statistics.

12 FINAL REPORT

A final report including clinical, bioanalytical, and statistical sections will be the responsibility of Syneos Health or a designee identified by the Sponsor and will be signed and approved by at least the Investigator.

In the event that the study is prematurely terminated, Syneos Health or a designee identified by the Sponsor will produce an abbreviated safety report. In such an event, raw data will not be submitted with the abbreviated report but will be archived at the clinical site, unless requested by the Sponsor.

13 REGULATORY CONSIDERATIONS AND QUALITY ASSURANCE

13.1 Independent Ethics Committee Approval of Protocol and Other Study Documents

The Investigator(s) agree to provide the IEC with all appropriate documents, including a copy of the protocol/amendments, ICFs, advertising text (if any), Investigator's Brochure (if any) and any other written information provided to study subjects. The trial will not begin until the Investigators have obtained the IEC favourable written approvals for the above-mentioned study documents. A properly executed written ICF shall be read, signed, and dated by each subject prior to entering the trial or prior to performing any study procedure. The original signed and dated ICF will be kept at Syneos Health and a copy will be given to the subject.

In the event that the protocol is amended, the revised protocol must be approved by the IEC prior to its implementation, unless the changes involve only logistical or administrative aspects of the trial. If a revised ICF is introduced during the study, each subject's further consent must be obtained. The new version of the ICF must be approved by the IEC, prior to subsequently obtaining each subject's consent.

The Investigator and the Sponsor's representative must sign the protocol and its amendments (if any) before initiating the study.

It is the Sponsor's responsibility to submit the protocol and its amendments (if any), and the ICFs to regulatory authorities when necessary.

13.2 Compliance

This study will be conducted in compliance with the protocol, GCP, and all applicable regulations, including the Federal Food, Drug and Cosmetic Act, U.S. applicable CFR (title 21), and any IEC requirements relative to clinical studies. The study will also be conducted in compliance with the recommendations laid down in the most recent version of the Declaration of Helsinki, with the exception that registration of such Phase 1 trials in a publicly accessible database is not mandatory. As required by the Canadian regulatory agency, a Clinical Trial Application (CTA) will be submitted before the beginning of the study and a No Objection Letter (NOL) must be received prior to dosing.

13.3 Quality Assurance Program

Syneos Health has established Quality Control (QC) and Quality Assurance (QA) systems with written SOPs to ensure that the study will be conducted and data will be generated, recorded, and reported in compliance with the protocol, GCP, and applicable regulatory requirements. A rigorous QC program is applied to ensure accuracy of all data and reports. QA oversees a complementary risk-based program of audits to assure compliance with applicable regulations and Syneos Health's prescriptive documentation.

13.4 Audits, Inspections and Monitoring

In accordance with the principles of GCP and GLP, the study may be inspected by regulatory authorities, the Sponsor and Syneos Health. The Sponsor is entitled to access information about the status of the study and to review the original documents of the study.

14 CONFIDENTIALITY AND RETENTION OF STUDY RECORDS

This document contains trade secrets and commercial information that is confidential and may not be disclosed to third parties. Persons to whom this study protocol is disclosed must be informed that all the information herein is confidential and may not be further divulged. These restrictions will apply as well to all future communications if deemed privileged or confidential. Publication of the study results may only be allowed with written permission from the Sponsor.

All information on a subject obtained during the conduct of the study will be kept confidential. Subjects will be identified by an anonymized identifier on all samples and study records provided to the Sponsor or designee. In compliance with ICH GCP, the Sponsor's authorized representatives, monitor(s), auditor(s), IEC, and regulatory authority(ies) will be granted direct access to the subject's original trial-related records for verification of clinical trial procedures and/or data, without violating the confidentiality of the subject, to the extent permitted by the applicable laws and regulations. Consent from the subject for disclosure of such information will be obtained in writing in the ICF. In addition, should a subject require medical care or hospitalization during the course of the study, the clinical site may contact the treating physician with the subject's consent, except that consent may not be requested if there is an emergency situation. If the results of the study are published, the subject's identity will remain confidential.

The clinical site will maintain adequate study records for 25 years after completion or termination of study. After this period, the Sponsor will be contacted to determine whether the study records will be forwarded to the Sponsor, destroyed or kept at the clinical site or another facility for a longer period of time at the Sponsor's expense.

15 REFERENCES

1 NURTEC ODT, Prescribing Information. Version revised on 02/27/2020. Drug Databases, FDA. Available online at: www.accessdata.fda.gov/scripts/cder/daf/

2 Rimegepant (BHV-3000). Investigator Brochure. Biohaven Pharmaceuticals, Inc. Edition No. 7.0 dated 25-Sep-2019.

3 NEORAL, Product Monograph. Version revised on January 9, 2015. Drug Product Database, Health Canada. Available online at: health-products.canada.ca/dpd-bdpp/index-eng.jsp

4 NEORAL, Prescribing Information. Version revised on 03/31/2015. Drug Databases, FDA. Available online at: www.accessdata.fda.gov/scripts/cder/daf/

5 In-house data. Syneos Health.

6 Quinidine Sulfate Tablets, USP 200 mg and 300 mg, Drug Label Information. NIH U.S. National Library of Medicine. Version revised on 10/24/2018. Available online at: dailymed.nlm.nih.gov/dailymed/index.cfm

7 Center for Drug Evaluation and Research (CDER), FDA. Clinical Drug Interaction Studies – Cytochrome P450 Enzyme- and Transporter-Mediated Drug Interactions. Guidance for Industry. January 2020.

8 FDA. US Food and Drug Administration - Drug Development and Drug Interactions: Table of Substrates, Inhibitors and Inducers.

9 Rebello, S., *et al*. Effect of cyclosporine on the pharmacokinetics of aliskiren in healthy subjects. Journal of clinical pharmacology. 2011. 51(11):1549-1560.

10 Aslanis, V., *et al*. Effect of cyclosporine coadministration on the pharmacokinetics of eltrombopag in healthy volunteers. Cancer chemotherapy and pharmacology. 2018. 82(5):847-855.

11 Bui, K., *et al*. The effect of quinidine, a strong P-glycoprotein inhibitor, on the pharmacokinetics and central nervous system distribution of naloxegol. Journal of clinical pharmacology. 2016. 56(4):497-505.

12 Kim, T.-E., *et al*. Effects of HM30181, a P-glycoprotein inhibitor, on the pharmacokinetics and pharmacodynamics of loperamide in healthy volunteers. British journal of clinical pharmacology. 2014. 78(3):556-564.

13 Sheehan, D.V., *et al*. Comparative Validation of the S-STS, the ISST-Plus, and the C-SSRS for Assessing the Suicidal Thinking and Behavior FDA 2012 Suicidality Categories. Innov Clin Neurosci. 2014. 11(9-10):32-46.

14 Sheehan, D.V., Giddens, J.M. and Sheehan, I.S. Status Update on the Sheehan-Suicidality Tracking Scale (S-STS), 2014. Innov Clin Neurosci. 2014. 11(9-10):93-140.
