# Supplementary figures and images for: P‐Glycoprotein and Breast Cancer Resistance Protein Transporter Inhibition by Cyclosporine and Quinidine on the Pharmacokinetics of Oral Rimegepant in Healthy Subjects
Source: Clin Pharmacol Drug Dev. 2022 Mar 19;11(7):889–97. doi: 10.1002/cpdd.1088 (PMC9311059; doi:10.1002/cpdd.1088)

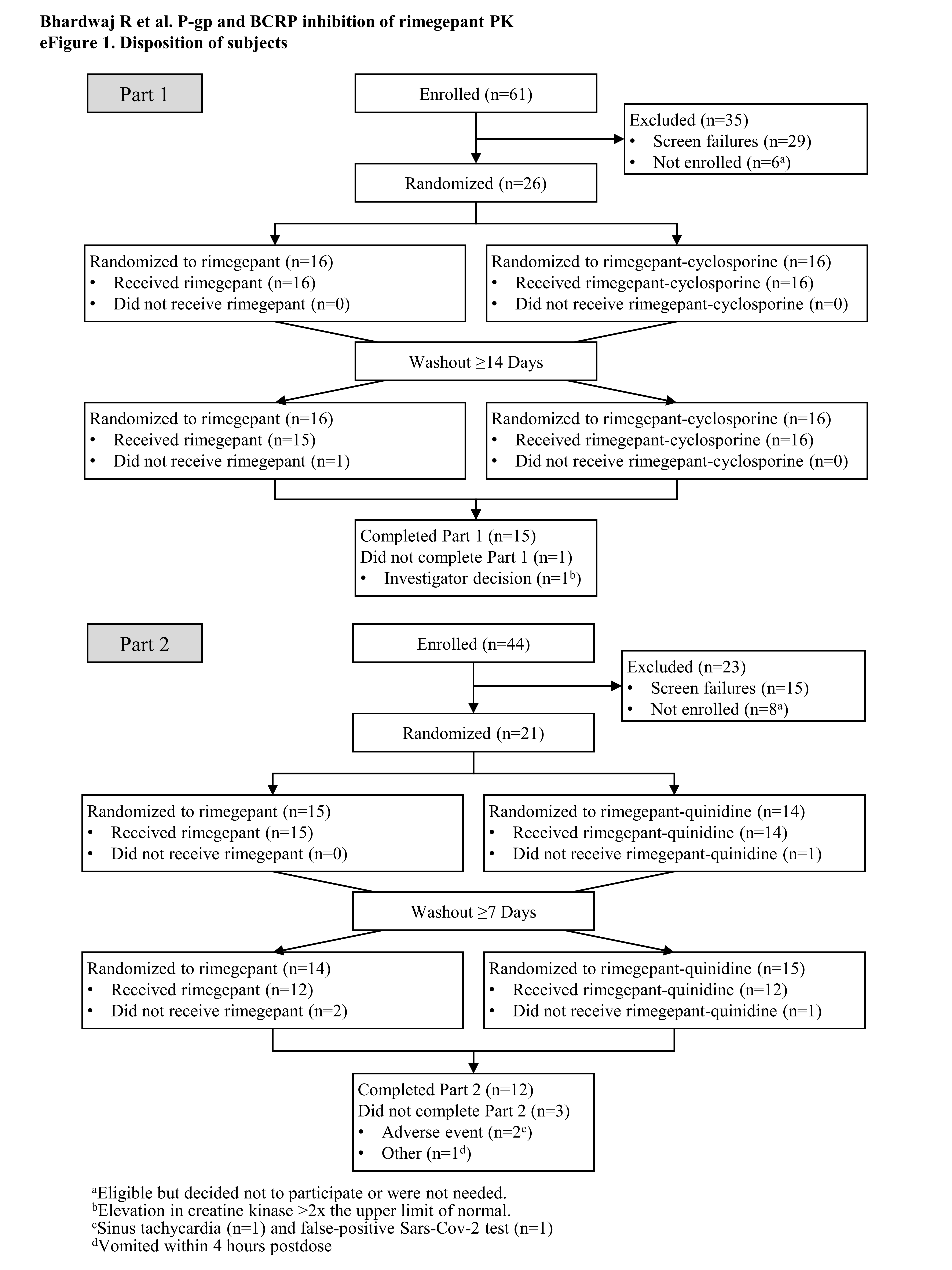

Supplement: Supplementary file 2 — Figure 2 [file CPDD-11-889-s001.tif]
